# A positive feedback loop between Flower and PI(4,5)P_2_ at periactive zones controls bulk endocytosis in Drosophila

**DOI:** 10.7554/eLife.60125

**Published:** 2020-12-10

**Authors:** Tsai-Ning Li, Yu-Jung Chen, Ting-Yi Lu, You-Tung Wang, Hsin-Chieh Lin, Chi-Kuang Yao

**Affiliations:** 1 Institute of Biological Chemistry, Academia SinicaTaipeiTaiwan; 2 Neuroscience Program of Academia Sinica, Academia SinicaTaipeiTaiwan; 3 Institute of Biochemical Sciences, College of Life Science, National Taiwan UniversityTaipeiTaiwan; Baylor College of MedicineUnited States; The University of Texas at AustinUnited States

**Keywords:** synaptic vesicle endocytosis, Ca2+ channel, PIP2, bulk endocytosis, periactive zone, *D. melanogaster*

## Abstract

Synaptic vesicle (SV) endocytosis is coupled to exocytosis to maintain SV pool size and thus neurotransmitter release. Intense stimulation induces activity-dependent bulk endocytosis (ADBE) to recapture large quantities of SV constituents in large endosomes from which SVs reform. How these consecutive processes are spatiotemporally coordinated remains unknown. Here, we show that Flower Ca^2+^ channel-dependent phosphatidylinositol 4,5-bisphosphate (PI(4,5)P_2_) compartmentalization governs control of these processes in *Drosophila*. Strong stimuli trigger PI(4,5)P_2_ microdomain formation at periactive zones. Upon exocytosis, Flower translocates from SVs to periactive zones, where it increases PI(4,5)P_2_ levels via Ca^2+^ influxes. Remarkably, PI(4,5)P_2_ directly enhances Flower channel activity, thereby establishing a positive feedback loop for PI(4,5)P_2_ microdomain compartmentalization. PI(4,5)P_2_ microdomains drive ADBE and SV reformation from bulk endosomes. PI(4,5)P_2_ further retrieves Flower to bulk endosomes, terminating endocytosis. We propose that the interplay between Flower and PI(4,5)P_2_ is the crucial spatiotemporal cue that couples exocytosis to ADBE and subsequent SV reformation.

## Introduction

Proper synaptic vesicle (SV) exocytosis dictates the robustness of brain activity. Coupling SV exocytosis with proper endocytosis is crucial for maintaining a balance of SV proteins at the release site, plasma membrane equilibrium, SV identity, and SV pool size (Chanaday et al., 2019; Haucke et al., 2011; Lou, 2018; Wu et al., 2014a). Currently, four modes of SV endocytosis are proposed. These differ in terms of stimulation intensity for their induction, formation, and molecular components (Haucke et al., 2011; Kononenko and Haucke, 2015; Wu et al., 2014a). Under mild neuronal stimulation, the SV partially fuses with the plasma membrane and reforms at the active zone, the so called ‘kiss and run’ mode. During clathrin-mediated endocytosis (CME), the SV fully collapses into the plasma membrane, followed by reformation of a single SV. Ultrafast endocytosis was also shown to recycle SVs at a sub-second timescale by forming a ~ 80 nm-sized bulk endosome predominantly at the edge of the active zone. SVs subsequently regenerate from this bulk endosome (Granseth et al., 2006; Watanabe et al., 2013a; Watanabe et al., 2013b; Zhu et al., 2009). High frequency stimulation and thus exocytosis could easily surpass the capacity of the three above-described endocytic mechanisms. It has therefore been proposed that activity-dependent bulk endocytosis (ADBE) has the necessary recapture capacity upon intense stimulation (Clayton et al., 2008; Soykan et al., 2017; Wu and Wu, 2007). This retrieval mode is elicited at the periactive zone to recapture large quantities of SV constituents via bulk endosome (~100–500 nm) formation, from which SVs subsequently reform. Hence, specific routes of SV recycling may fit the specific demands of a wide range of neuronal activities at the synapse. However, how stimulation intensity dictates the choice between these different endocytic modes is not well understood.

ADBE has been documented in many different types of neurons in invertebrates and vertebrates (Clayton et al., 2008; Heerssen et al., 2008; Heuser and Reese, 1973; Holt et al., 2003; Kasprowicz et al., 2008; Kittelmann et al., 2013; Miller and Heuser, 1984; Richards et al., 2000; Soykan et al., 2017; Stevens et al., 2012; Vijayakrishnan et al., 2009; Wenzel et al., 2012; Wu and Wu, 2007; Yao et al., 2017). Although the precise mechanism regulating ADBE is not known, multiple lines of evidence suggest that actin polymerization may serve as the membrane invagination force responsible for generating bulk endosomes (Gormal et al., 2015; Holt et al., 2003; Kokotos and Low, 2015; Nguyen et al., 2012; Richards et al., 2004; Soykan et al., 2017; Wu et al., 2016). Furthermore, phosphatidylinositol metabolism has also been implicated in controlling this recycling mode (Gaffield et al., 2011; Holt et al., 2003; Richards et al., 2004; Vijayakrishnan et al., 2009). Importantly, loss of Synaptojanin, the major phosphatidylinositol 4,5-bisphosphate (PI(4,5)P_2_) catalytic enzyme in neurons (Tsujishita et al., 2001), leads to reduced SV endocytosis elicited by intense stimulation, presumably by affecting ADBE (Mani et al., 2007). PI(4,5)P_2_ is known to promote actin polymerization by activating a number of actin modulators (Janmey et al., 2018). PI(4,5)P_2_ is clustered to form microdomains upon the demand for diverse cellular functions (Aoyagi et al., 2005; Chen et al., 2015; Honigmann et al., 2013; Kabeche et al., 2015; Mu et al., 2018; Picas et al., 2014; Riggi et al., 2018; van den Bogaart et al., 2011). It has also been reported that formation of PI(4,5)P_2_ microdomains precedes actin polymerization during a process reminiscent of ADBE in neurosecretory cells (Gormal et al., 2015). Thus, subcellular compartmentalization of PI(4,5)P_2_ may provide the spatial information dictating where bulk membranes will invaginate. However, the mechanism initiating the formation of the PI(4,5)P_2_ microdomains is unknown.

Coordinated SV protein and membrane retrieval plays an important role in maintaining the identity of newly formed SVs during SV recycling (Kaempf and Maritzen, 2017; McMahon and Boucrot, 2011; Saheki and De Camilli, 2012; Traub and Bonifacino, 2013). It has been well documented that proper sorting of SV proteins to the nascent SV is achieved in CME by the cooperative action of PI(4,5)P_2_ and adaptor protein complexes (Saheki and De Camilli, 2012; Traub and Bonifacino, 2013). Recent studies have also revealed a distinct sorting mechanism that retrieves selective SV cargoes to the bulk endosome via ADBE (Kokotos et al., 2018; Nicholson-Fish et al., 2015). Several lines of evidence further suggest that clathrin and adaptor protein complexes are required for reforming SVs from the bulk endosome (Cheung and Cousin, 2012; Glyvuk et al., 2010; Kokotos et al., 2018; Kononenko et al., 2014; Park et al., 2016). A dynaminI/dynaminIII/clathrin-independent mechanism has also been reported as being involved in this process (Wu et al., 2014c). Thus, during SV regeneration via ADBE, multiple protein sorting steps may be required to ensure that the SVs harbor the proper compositions of lipids and proteins, thereby endowing specific release probabilities in relation to other modes of endocytosis (Cheung et al., 2010; Hoopmann et al., 2010; Nicholson-Fish et al., 2015; Silm et al., 2019). The mechanism by which protein sorting and membrane retrieval are coordinated in this process remains to be explored.

Here, we show that, upon intense stimulation, PI(4,5)P_2_ is compartmentalized into microdomains at periactive zones in the synaptic boutons of *Drosophila* larval neuromuscular junctions (NMJs). Blockade of PI(4,5)P_2_ microdomain formation diminishes ADBE and SV reformation from the bulk endosome. Increased intracellular Ca^2+^ and SV exocytosis are prerequisites for initiating ADBE (Morton et al., 2015; Wu and Wu, 2007). We have previously shown that Flower (Fwe), a SV-associated Ca^2+^ channel, regulates both CME and ADBE, and that its channel activity is strongly activated upon intense stimulation to elicit ADBE (Yao et al., 2017). We show that Fwe initiates a positive feedback loop upon PI(4,5)P_2_ increase to ensure the formation of PI(4,5)P_2_ microdomains and thus trigger ADBE and subsequent SV reformation. Intriguingly, PI(4,5)P_2_ also participates in retrieval of Fwe to the bulk endosome, thereby stopping membrane recycling. Hence, spatiotemporal interplays between Flower and PI(4,5)P_2_ coordinate the retrieval of SV cargos and membranes, coupling exocytosis to ADBE and subsequent SV reformation.

## Results

### Intense neuronal activity induces formation of PI(4,5)P_2_ microdomains at the presynaptic periactive zone of *Drosophila* synapses

**Figure 1.**
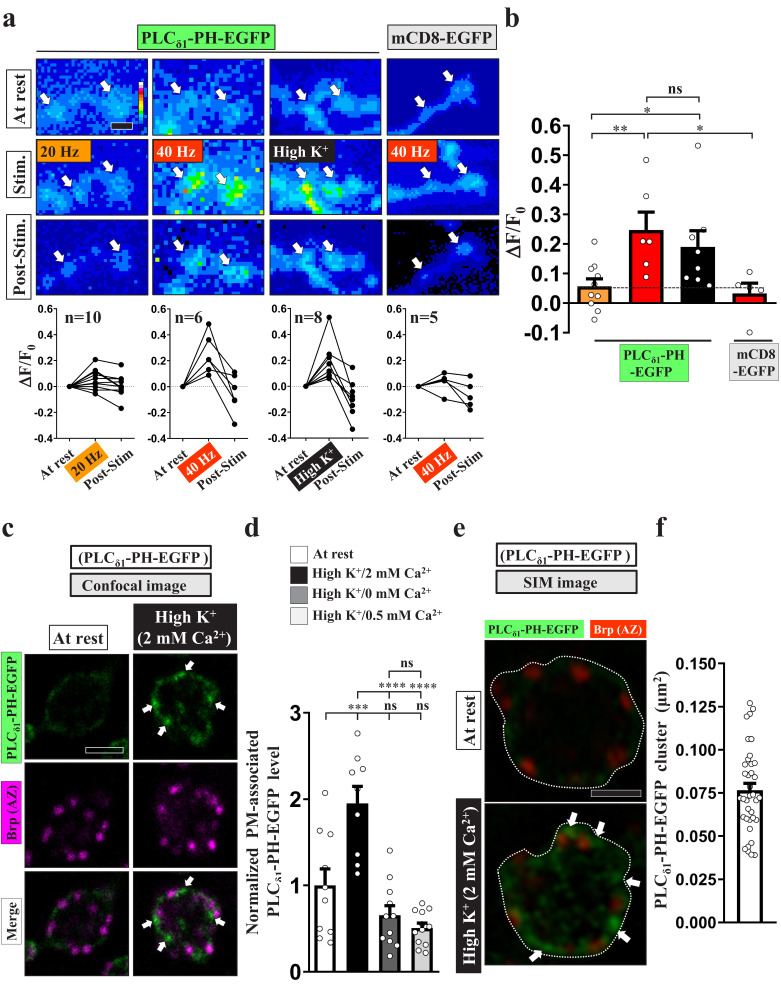
PI(4,5)P_2_ forms microdomains at periactive zones under conditions of intense stimulation. (**a–b**) Increased fluorescence of PLC_δ1_-PH-EGFP but not mCD8-GFP in NMJ boutons upon intense stimulation. (**a**) (Top) Live images of the boutons (arrows) expressing *UAS-PLC_δ1_-PH-EGFP* or *UAS-mCD8-GFP*. The larvae were reared at 25°C. Electrical (20 or 40 Hz) or chemical (90 mM K^+^) stimulation was conducted in a 2 mM-Ca^2+^ solution for 3 min (electrical) or 5 min (chemical) and then rested in 0 mM Ca^2+^ and 5 mM K^+^. Snapshot images taken before stimulation, at the third (electrical) and fifth (chemical) min of stimulation, and after stimulation. (Bottom) Traces of probe fluorescence for single boutons. The number of boutons imaged (N). (**b**) Quantification data for EGFP fluorescence change. The resting fluorescence level (F_0_). Fluorescence change evoked by stimulation (ΔF). (**c–f**) PLC_δ1_-PH-EGFP enrichment at periactive zones is dependent on Ca^2+^ upon intense stimulation. Single-plane confocal (**c**) or SIM (**e**) images of the boutons expressing PLC_δ1_-PH-EGFP. The larvae were reared at 25°C. The boutons subjected to high K^+^/2 mM Ca^2+^ (10 min stimulation of 90 mM K^+^/2 mM Ca^2+^), high K^+^/0 mM Ca^2+^ (10 min stimulation of 90 mM K^+^/0 mM Ca^2+^), or high K^+^/0.5 mM Ca^2+^ (1 min stimulation of 90 mM K^+^/0.5 mM Ca^2+^) treatments were fixed immediately and immunostained for PLC_δ1_-PH-EGFP (green) and Bruchpilot (Brp) [an active zone scaffold protein; magenta (c), red (e)]. The PLC_δ1_-PH-EGFP-enriched puncta (Arrows). (**d**) Quantification data for PLC_δ1_-PH-EGFP staining intensities, normalized to the value of the resting condition. (**f**) Average area of PLC_δ1_-PH-EGFP clusters in individual boutons, as measured by SIM. Individual data values are shown in graphs. p values: ns, not significant; *p<0.05; **p<0.01; ***p<0.001; ****p<0.0001. Mean ± SEM. Scale bar: 1 µm (**e**), 2 µm (**a, c**). Statistics: one-way ANOVA with Tukey’s post hoc test (**b, d**). Figure 1—source data 1.Source data for Figure 1.

To investigate the dynamics of PI(4,5)P_2_ in the presynaptic compartment, we expressed a GFP fusion protein of the pleckstrin homology (PH) domain of PLC_δ1_ (PLC_δ1_-PH-EGFP) in synaptic boutons of *Drosophila* larval NMJs using *nSyb-GAL4*, a pan-neuronal driver. PLC_δ1_-PH-EGFP binds to PI(4,5)P_2_ with high affinity and is widely used to label subcellular compartments in which PI(4,5)P_2_ is enriched (Chen et al., 2014; Khuong et al., 2010). We delivered 20 Hz stimuli for three minutes to synaptic boutons in a 2 mM extracellular Ca^2+^ solution and performed live imaging in the third minute. Consecutive snapshot images were taken before stimulation, during the third minute of stimulation, and after stimulation. As shown in Figure 1a–b, we observed a very subtle increase in PLC_δ1_-PH-EGFP fluorescence in individual boutons (white arrows), similar to findings of a previous study (Verstreken et al., 2009). However, when we raised the stimulus intensity to 40 Hz, we recorded a robust increase in fluorescence relative to a GFP fusion protein of the plasma membrane-integrated mCD8 domain (*UAS-mCD8-GFP*). Fluorescence signals rapidly returned to basal levels within tens of seconds when the stimuli were removed. We have previously documented that treatment with 40 Hz electric pulses or 90 mM high KCl solution can cause comparable stimulation intensities in *Drosophila* NMJ boutons (Yao et al., 2017). High K^+^ treatment also increased the fluorescence signal of PLC_δ1_-PH-EGFP. No increase in the presynaptic protein level of PLC_δ1_-PH-EGFP was found under this condition (Figure 1—figure supplement 1a–b), arguing that this stimulation does not induce protein synthesis. These results suggest that, in response to intense stimulation, PLC_δ1_-PH-EGFP is redistributed and concentrated to PI(4,5)P_2_-enriched subdomains of the plasma membrane, thereby enhancing the overall fluorescence.

**Figure 1—figure supplement 1.**
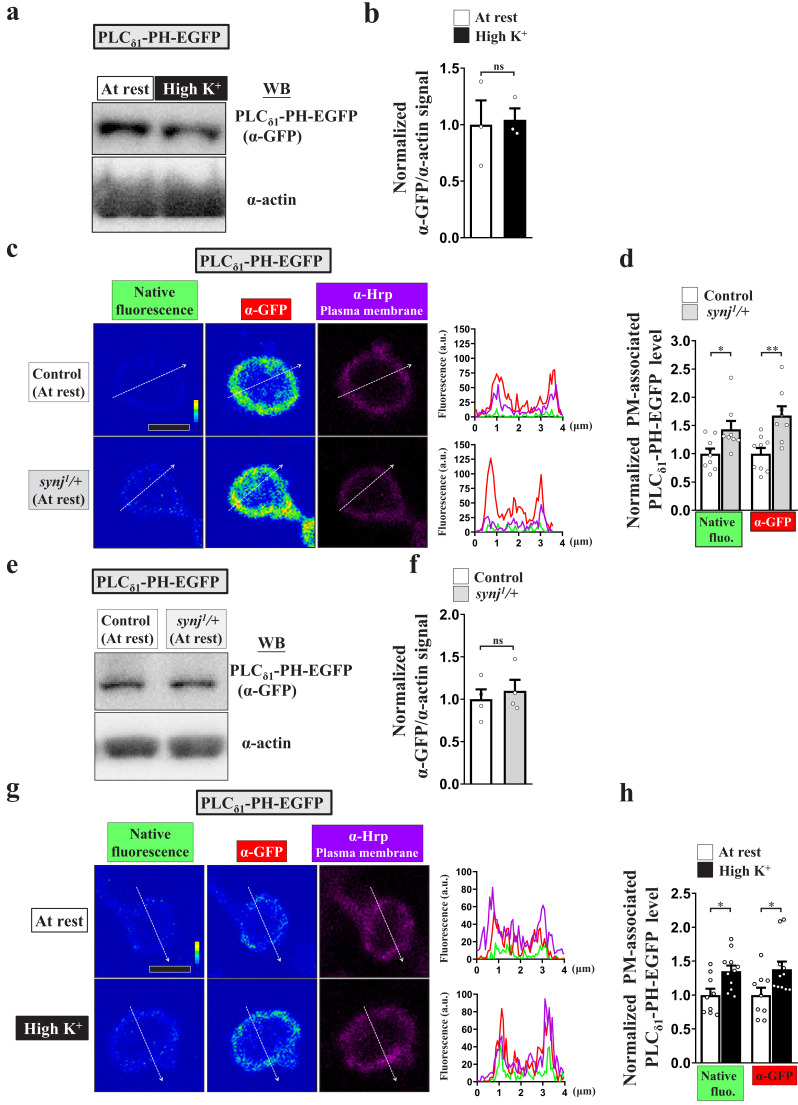
The change in PLC_δ1_-PH-EGFP fluorescence is responsible for an increase in the PI(4,5)P_2_ level. (**a–b**) The protein expression level of PLC_δ1_-PH-EGFP was not changed upon high K^+^ stimulation. (**a**) Larval fillets expressing *UAS-PLC_δ1_-PH-EGFP* using *nSyb-GAL4* were dissected, and the brain and ventral nerve cord were removed. The samples were subjected to the resting condition (10 min incubation of 5 mM K^+^/0 mM Ca^2+^) or high K^+^ stimulation (10 min stimulation of 90 mM K^+^/2 mM Ca^2+^), and then immediately lysed in 1x SDS sample buffer. The lysate of a single larva was loaded in each lane of the western blot, and α-GFP and α-actin were used to detect presynaptic levels of PLC_δ1_-PH-EGFP or to serve as loading control, respectively. (**b**) Quantification data for the ratio of α-GFP to α-actin blotting signal, normalized to the value of the resting condition. (**c–f**) Reducing Synj levels increased the levels of PI(4,5)P_2_ and thus association of PLC_δ1_-PH-EGFP on the plasma membrane, but it did not increase the protein expression level of PLC_δ1_-PH-EGFP in synaptic boutons. (**c–d**) Single-plane confocal images of NMJ boutons neuronally expressing *UAS-PLC_δ1_-PH-EGFP* using *nSyb-GAL4* in the wild-type control or *synj^1^*/+ lines. The boutons were subjected to the resting condition (10 min incubation of 5 mM K^+^/0 mM Ca^2+^), followed by chemical fixation and α-GFP immunostaining. Line plot profiles of absolute fluorescence units (a.u.) of native fluorescence or α-GFP immunostaining signal of PLC_δ1_-PH-EGFP are shown at right. α-Hrp immunostaining was used to outline the presynaptic plasma membrane. (**d**) Quantification data for the plasma membrane-associated PLC_δ1_-PH-EGFP fluorescence or immunostaining intensities, normalized to the value of wild-type boutons. (**e–f**) Brain-removed larval fillets expressing *PLC_δ1_-PH-EGFP* were subjected to western blot using α-GFP and α-actin antibodies. Quantification data for the ratio of α-GFP to α-actin blotting signal, normalized to the value of the wild-type control fillets (**f**). (**g–h**) High K^+^ stimulation elevated plasma membrane localization but not protein expression level of PLC_δ1_-PH-EGFP. (**g**) Single-plane confocal images of NMJ boutons neuronally expressing *UAS-PLC_δ1_-PH-EGFP* using *nSyb-GAL4*. The boutons were subjected to the resting condition (10 min incubation of 5 mM K^+^/0 mM Ca^2+^) or high K^+^ stimulation (10 min stimulation of 90 mM K^+^/2 mM Ca^2+^), followed by chemical fixation and α-GFP immunostaining. Line plot profiles of absolute fluorescence units (a.u.) of native fluorescence or α-GFP immunostaining signal of PLC_δ1_-PH-EGFP are shown at right. α-Hrp immunostaining was used to outline the presynaptic plasma membrane. (**h**) Quantification data for the plasma membrane-associated PLC_δ1_-PH-EGFP fluorescence or immunostaining intensities, normalized to the value of the resting condition. Individual data values are shown in graphs. p values: ns, not significant; *p<0.05; **p<0.01. Mean ± SEM. Scale bar: 2 µm (**c, g**). Statistics: Student *t*-test. Figure 1—figure supplement 1—source data 1.Source data for Figure 1—figure supplement 1.

**Figure 1—figure supplement 2.**
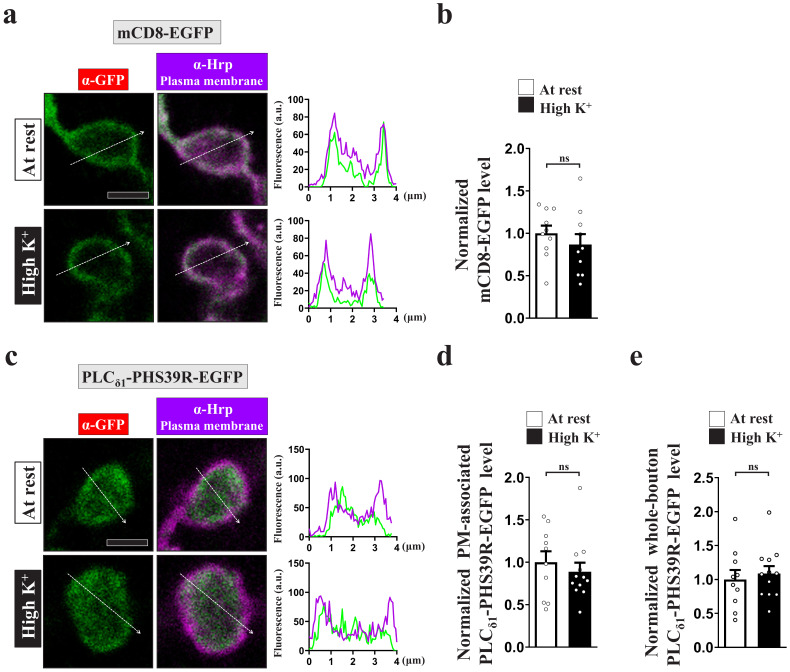
The distribution of mCD8-EGFP and PLC_δ1_-PHS39R-EGFP remains unchanged upon intense stimulation. Single-plane confocal images of NMJ boutons neuronally expressing *UAS-mCD8-EGFP* (**a**) or *UAS-PLC_δ1_-PHS39R-EGFP* (**c**) using *nSyb-GAL4*. The boutons were subjected to the resting condition (10 min incubation of 5 mM K^+^/0 mM Ca^2+^) or high K^+^ stimulation (10 min stimulation of 90 mM K^+^/2 mM Ca^2+^), followed by chemical fixation and α-GFP immunostaining. Line plot profiles of absolute fluorescence units (a.u.) of α-GFP immunostaining signal of both probes are shown at right. α-Hrp immunostaining (magneta) was used to outline the presynaptic plasma membrane. mCD8-EGFP (green) was exclusively localized in the plasma membrane, whereas PLC_δ1_-PHS39R-EGFP (green) was mainly expressed in the cytosol. Quantification data for immunostaining intensities of the plasma membrane-associated mCD8-EGFP (**b**), the plasma membrane-associated PLC_δ1_-PHS39R-EGFP (**d**), or the whole-bouton PLC_δ1_-PHS39R-EGFP (**e**), normalized to the value of the resting condition. Individual data values are shown in graphs. p values: ns, not significant. Mean ± SEM. Scale bar: 2 µm (**a, c**). Statistics: Student *t*-test. Figure 1—figure supplement 2—source data 1.Source data for Figure 1—figure supplement 2.

**Figure 1—figure supplement 3.**
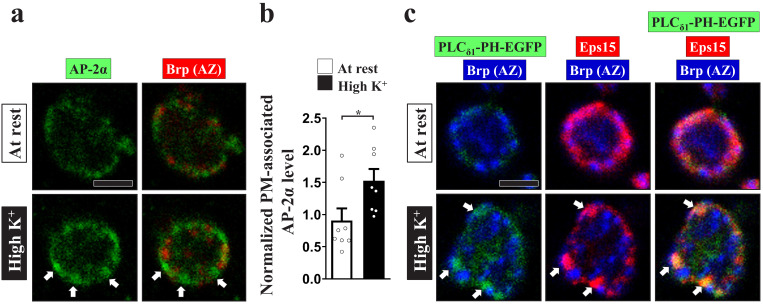
PI(4,5)P_2_ is increased at the periactive zone upon intense stimulation. (**a–b**) AP-2 complex was actively recruited to the plasma membrane after the boutons were intensely stimulated. (**a**) Single-plane confocal images of NMJ boutons stained with α-AP-2α. The boutons were subjected to the resting condition (10 min incubation of 5 mM K^+^/0 mM Ca^2+^) or high K^+^ stimulation (10 min stimulation of 90 mM K^+^/2 mM Ca^2+^), followed by chemical fixation and α-AP-2α immunostaining (green). Active zones were labeled with Brp staining (red). An increase in AP-2α immunostaining signal was observed at the periactive zones (arrows). (**b**) Quantification data for the plasma membrane-associated α-AP-2α immunostaining intensities, normalized to the value of the resting condition. Individual data values are shown in graphs. p values: *p<0.05. Mean ± SEM. Scale bar: 2 µm. Statistics: Student *t*-test. (**c**) Upon high K^+^ stimulation, PLC_δ1_-PH-EGFP accumulated in PI(4,5)P_2_-enriched microdomains at the periactive zones. Single-plane confocal images of NMJ boutons neuronally expressing *UAS-PLC_δ1_-PH-EGFP* using *nSyb-GAL4*. The boutons were subjected to the resting condition (10 min incubation of 5 mM K^+^/0 mM Ca^2+^) or high K^+^ stimulation (10 min stimulation of 90 mM K^+^/2 mM Ca^2+^), followed by chemical fixation and immunostaining. Arrows indicate that Eps15 (red) was concentrated at the periactive zone and colocalized with PLC_δ1_-PH-EGFP (green) stained with α-GFP after intense stimulation. Brp (blue) labels active zones. Scale bar: 2 µm. Figure 1—figure supplement 3—source data 1.Source data for Figure 1—figure supplement 3.

To characterize the subcellular distribution of the stimulus-dependent PI(4,5)P_2_ induction, we conducted a chemical fixation protocol whereby the NMJ boutons were fixed at rest or immediately after high K^+^ stimulation and then immunostained for PLC_δ1_-PH-EGFP using an α-GFP antibody to enhance the signal for high-resolution microscopic imaging. Confocal images revealed that, for neurons at rest, native PLC_δ1_-PH-EGFP fluorescence was weakly detected on the presynaptic plasma membrane labeled by α-Hrp staining, with some additional fluorescent signal being dispersed in the cytosol (Figure 1—figure supplement 1c). We then elevated PI(4,5)P_2_ on the plasma membrane by removing a copy of *synaptojanin* (*synj*), which encodes the major neuronal PI(4,5)P_2_ phosphatase (Tsujishita et al., 2001; Verstreken et al., 2003). Consistent with previous studies (Chen et al., 2014; Verstreken et al., 2009), this reduction in Synj levels enhanced PLC_δ1_-PH-EGFP fluorescence (Figure 1—figure supplement 1c–d). In this context, we did not observe a significant change in protein expression of PLC_δ1_-PH-EGFP in the presynaptic compartment (Figure 1—figure supplement 1e–f). By using α-GFP immunostaining, these PLC_δ1_-PH-EGFP signals could be faithfully amplified (Figure 1—figure supplement 1c–d). Therefore, this immunostaining approach was subsequently used to monitor presynaptic PI(4,5)P_2_ levels.

Next, we stimulated the boutons expressing PLC_δ1_-PH-EGFP with 90 mM K^+^ and 2 mM Ca^2+^ for 10 min. Similar to our live-imaging results, PLC_δ1_-PH-EGFP signals were significantly increased on the presynaptic plasma membrane (Figure 1—figure supplement 1g–h). In particular, we observed high-level induction of PLC_δ1_-PH-EGFP puncta (Figure 1c–d). To assess the possibility that chemical fixation may have altered membrane properties and consequently plasma membrane PLC_δ1_-PH-EGFP clustering upon intense stimulation, we further examined the distributions of mCD8-GFP and PLC_δ1_-PHS39R-EGFP, a PI(4,5)P_2_-binding mutant (Khuong et al., 2010; Verstreken et al., 2009). There was no obvious change in the plasma membrane pattern of mCD8-GFP in fixed boutons stimulated with high K^+^ (Figure 1—figure supplement 2a–b). Unlike PLC_δ1_-PH-EGFP, PLC_δ1_-PHS39R-EGFP was found mainly in the cytosol at rest. After intense stimulation, immunostaining signals of PLC_δ1_-PHS39R-EGFP did not accumulate on the plasma membrane (Figure 1—figure supplement 2c–d) and were not increased within the boutons (Figure 1—figure supplement 2e). Thus, clustering of PLCδ1-PH-EGFP on the plasma membrane upon stimulation results from an increase in local PI(4,5)P_2_ concentration, although a potential effect of chemical fixation, if any, on PLC_δ1_-PH-EGFP clustering cannot be excluded. To assess potential dominant-negative effects of PI(4,5)P_2_ binding by PLC_δ1_-PH-EGFP on the stimulation-induced accumulation of PI(4,5)P_2_, we further examined the recruitment of the AP-2 complex by PI(4,5)P_2_. Similar to PLC_δ1_-PH-EGFP, levels of the α subunit of the AP-2 complex (AP-2α) were also increased on the plasma membrane upon high K^+^ stimulation (Figure 1—figure supplement 3a–b). Together, these data indicate that intense stimulation can promote the formation of PI(4,5)P_2_ microdomains.

We noted that the induced PI(4,5)P_2_ microdomains were primarily sited at periactive zones marked by activity-dependent localization of Eps15 (Figure 1c; Figure 1—figure supplement 3c; Koh et al., 2007; Winther et al., 2013). Periactive zones are known hot-spots for ADBE (Chanaday et al., 2019; Kononenko and Haucke, 2015; Wu et al., 2014a). By using structured illumination microscopy (SIM), we estimated that the average size of these PI(4,5)P_2_ microdomains formed after stimulation is ~300 nm in diameter (Figure 1e–f) (0.07655 ± 0.004 μm^2^, mean ± S.E.M., n = 37 boutons).

Next, to determine the Ca^2+^ dependence of the PI(4,5)P_2_ microdomains, we stimulated the boutons in a solution of 90 mM K^+^ and 0 mM Ca^2+^, which resulted in failure to induce PI(4,5)P_2_ microdomain formation (Figure 1d). We obtained a similar result using 1 min stimulation of 90 mM K^+^ and 0.5 mM Ca^2+^ (Figure 1d), which was previously shown to primarily elicit CME but not ADBE (Yao et al., 2017). Hence, these results suggest that intense stimulation can elicit Ca^2+^-driven compartmentalization of PI(4,5)P_2_ at the periactive zone in synaptic boutons of *Drosophila* NMJs.

### PI(4,5)P_2_ microdomains are involved in ADBE initiation and SV reformation from bulk endosomes

**Figure 2.**
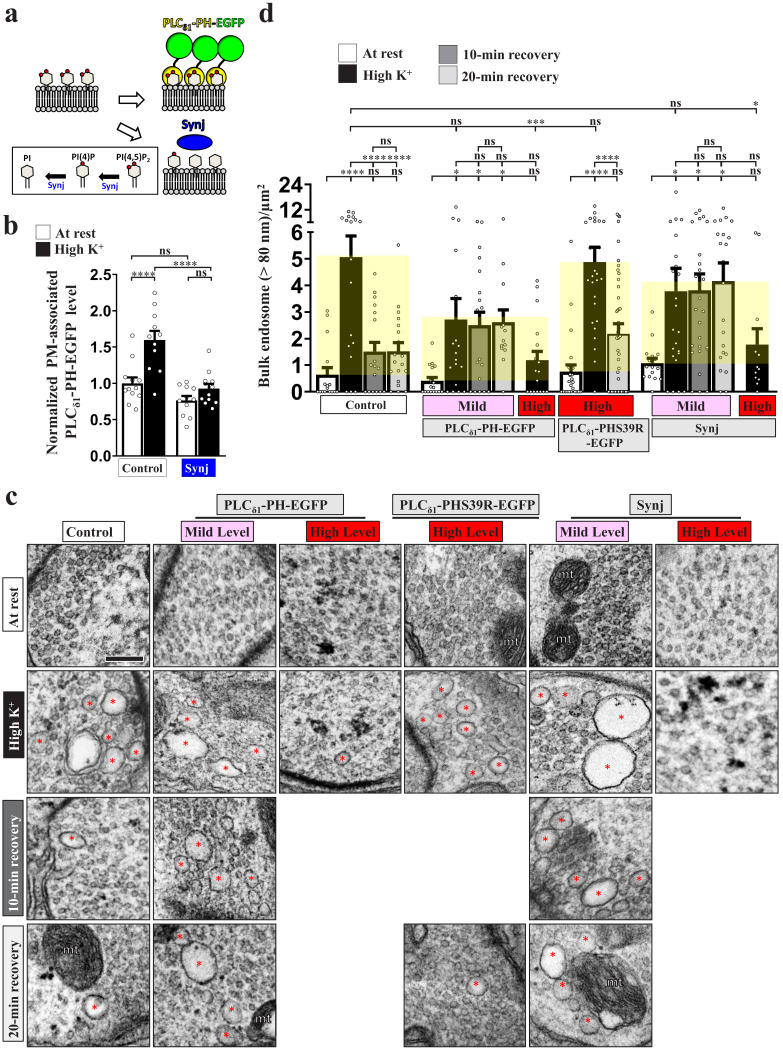
PI(4,5)P_2_ microdomains drive ADBE and SV reformation from bulk endosomes. Reducing PI(4,5)P_2_ availability suppresses ADBE and subsequent SV reformation. (**a**) A schematic for PI(4,5)P_2_ suppression by PLC_δ1_-PH-EGFP or Synj expression. (**b**) Expression of Synj reduced presynaptic plasma membrane PI(4,5)P_2_ upon high K^+^ treatment. The boutons co-expressing *UAS-PLC_δ1_-PH-EGFP* with *UAS-RFP* (control) or *UAS-synj* using *nSyb-GAL4* were reared at 25°C and subjected to resting condition (10 min incubation of 5 mM K^+^/0 mM Ca^2+^) or high K^+^ stimulation (10 min stimulation of 90 mM K^+^/2 mM Ca^2+^), followed by α-GFP immunostaining. Single-plane confocal images of the boutons are shown in Figure 2—figure supplement 1c. Quantification data for PLC_δ1_-PH-EGFP staining intensity are shown, normalized to the value of the resting condition of controls. (**c**) TEM images of the boutons of controls (*nSyb-GAL4/+* at 29°C), mild PLC_δ1_-PH-EGFP expression (*nSyb-GAL4/UAS-PLC_δ1_-PH-EGFP* at 25°C), high PLC_δ1_-PH-EGFP expression (*nSyb-GAL4/UAS-PLC_δ1_-PH-EGFP* at 29°C), high PLC_δ1_-PHS39R-EGFP expression (*nSyb-GAL4/UAS-PLC_δ1_-PHS39R-EGFP* at 29°C), mild Synj expression (*nSyb-GAL4/UAS-synj* at 25°C), or high Synj expression (*nSyb-GAL4/UAS-synj* at 29°C). At rest (10 min incubation of 5 mM K^+^/0 mM Ca^2+^). High K^+^ (10 min stimulation of 90 mM K^+^/2 mM Ca^2+^). 10 min recovery (10 min stimulation of 90 mM K^+^/2 mM Ca^2+^, followed by 10 min incubation of 5 mM K^+^/0 mM Ca^2+^). 20 min recovery (10 min stimulation of 90 mM K^+^/2 mM Ca^2+^, followed by 20 min incubation of 5 mM K^+^/0 mM Ca^2+^). Bulk endosomes (>80 nm in diameter, red asterisks). Mitochondria (mt). Quantification data for total number of bulk endosomes per bouton area (**d**). Individual data values are shown in graphs. p values: ns, not significant; *p<0.05; **p<0.01; ***p<0.001; ****p<0.0001. Mean ± SEM. Scale bar: 500 nm. Statistics: one-way ANOVA with Tukey’s post hoc test. Figure 2—source data 1.Source data for Figure 2.

Next, we investigated the function of PI(4,5)P_2_ microdomains in ADBE. High K^+^ treatment is widely used to trigger ADBE in a broad range of synapses (Akbergenova and Bykhovskaia, 2009; Clayton et al., 2008; Jin et al., 2019; Stevens et al., 2012; Vijayakrishnan et al., 2009; Wu and Wu, 2007; Wu et al., 2014c). To measure ADBE, we induced ADBE with 90 mM K^+^ and 2 mM Ca^2+^ for 10 min and then conducted transmission electron microscopy (TEM). ADBE was evoked in wild-type control boutons under these conditions and generated the formation of bulk endosomes (red asterisks, defined as >80 nm in diameter) (Figure 2c–d). To suppress the function of PI(4,5)P_2_, we expressed PLC_δ1_-PH-EGFP, anticipating that binding of the PLC_δ1_-PH domain to PI(4,5)P_2_ would restrict availability of PI(4,5)P_2_ to its effectors and metabolic enzymes (Figure 2a; Khuong et al., 2013). By using the *GAL4/UAS* system, we were able to adjust expression levels of the PLC_δ1_-PH domain via temperature manipulation (Brand and Perrimon, 1993; D'Avino and Thummel, 1999; Wilder, 2000). When we neuronally expressed PLC_δ1_-PH-EGFP using *nSyb-GAL4* and grew larvae at 25°C, we found that mild expression of PLC_δ1_-PH-EGFP had a mild inhibitory effect on ADBE induction relative to wild-type control boutons (Figure 2c–d). In contrast, when larvae were grown at 29°C to effect a two-fold increase in PLC_δ1_-PH-EGFP expression (Figure 2—figure supplement 1a–b), ADBE was almost completely abolished (Figure 2c–d). However, when larvae expressing PLC_δ1_-PHS39R-EGFP were grown at 29°C, ADBE was not suppressed by the mutant protein (Figure 2c–d).

**Figure 2—figure supplement 1.**
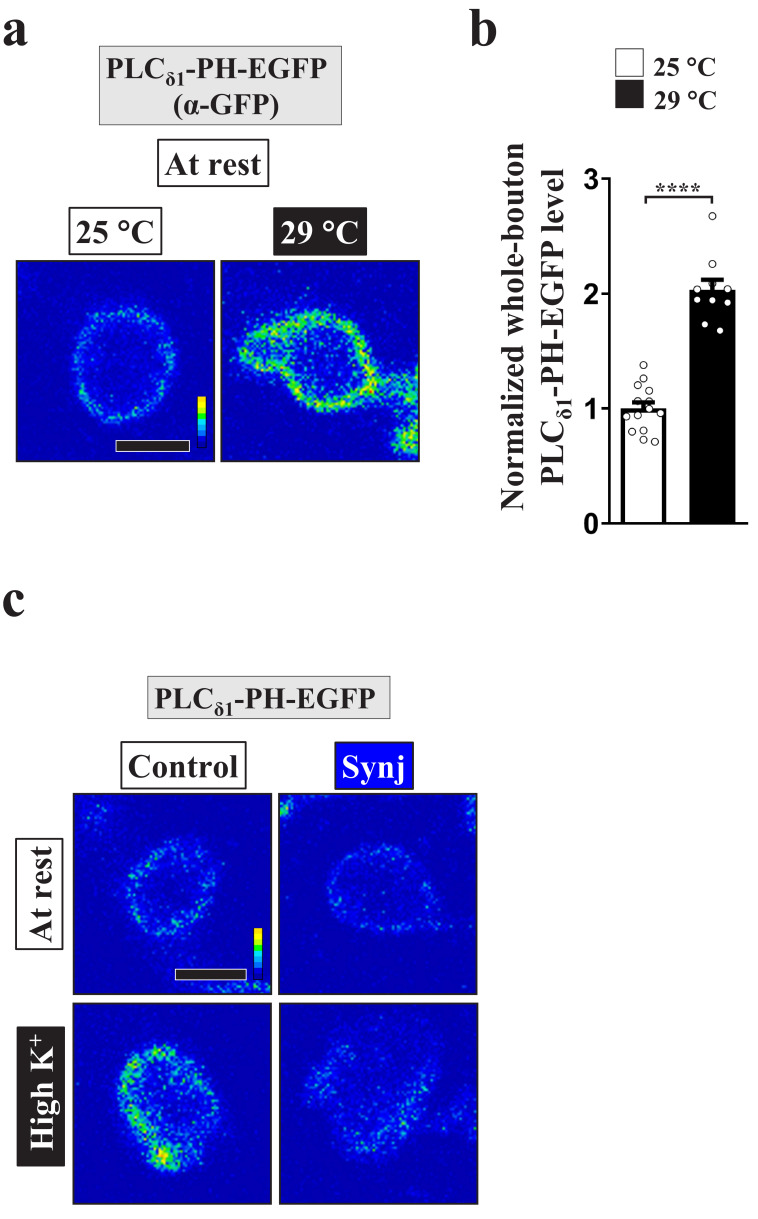
Blockade of the PI(4,5)P_2_ microdomains by expressing PLC_δ1_-PH-EGFP and Synj. (**a**) Increasing rearing temperature from 25°C to 29°C elevated PLC_δ1_-PH-EGFP expression. Single-plane confocal images of NMJ boutons neuronally expressing *UAS-PLC_δ1_-PH-EGFP* using *nSyb-GAL4*. The boutons were situated at rest and immunostained with α-GFP. (**b**) Quantification data for the whole-bouton PLC_δ1_-PH-EGFP immunostaining intensities, normalized to the value for the 25°C condition. Individual data values are shown in graphs. p values: ****p<0.0001. Mean ± SEM. Scale bar: 2 µm. Statistics: Student *t*-test. (**c**) Synj expression suppressed PI(4,5)P_2_ microdomain formation. Single-plane confocal images of NMJ boutons co-expressing *UAS-PLC_δ1_-PH-EGFP* with *UAS-RFP* control or *UAS-Synj* at 25°C. The boutons were subjected to the resting condition (10 min incubation of 5 mM K^+^/0 mM Ca^2+^) or high K^+^ stimulation (10 min stimulation of 90 mM K^+^/2 mM Ca^2+^), followed by chemical fixation and α-GFP immunostaining. Scale bar: 2 µm. Figure 2—figure supplement 1—source data 1.Source data for Figure 2—figure supplement 1.

**Figure 2—figure supplement 2.**
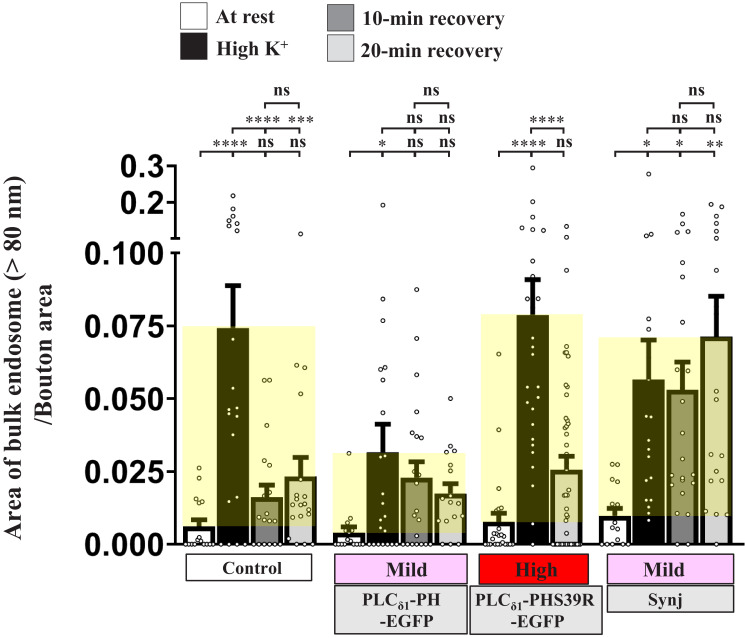
Blockade of the PI(4,5)P_2_ microdomains abolishes SV reformation from the bulk endosome. Quantification data for the ratio of total area of bulk endosomes to bouton area. Indicated genotypes were assessed. Representative TEM images are shown in Figure 2c. Individual data values are shown in graphs. p values: ns, not significant; *p<0.05; **p<0.01; ***p<0.001; ****p<0.0001. Mean ± SEM. Statistics: one-way ANOVA with Tukey’s post hoc test. Figure 2—figure supplement 2—source data 1.Source data for Figure 2—figure supplement 2.

Synj comprises a central 5-phosphatase domain that specifically dephosphorylates the 5' position of PI(4,5)P_2_ to produce PI(4)P (McPherson et al., 1996; Woscholski et al., 1997). In addition, an N-terminal Sac1 domain converts several phosphatidylinositides—including PI(3,5)P_2_, PI(3)P, and PI(4)P—to PI (Figure 2a; Guo et al., 1999). Indeed, overexpression of Synj reduced the formation of the PI(4,5)P_2_ microdomains induced by high K^+^ (Figure 2b; Figure 2—figure supplement 1c). Importantly, overexpression of Synj resulted in dosage-dependent inhibition of ADBE (Figure 2c–d), similar to the effect of increasing levels of the PLC_δ1_-PH domain. Together, these data suggest that the PI(4,5)P_2_ microdomains initiate ADBE to control SV membrane retrieval upon intense stimulation.

SVs regenerate from bulk endosomes within minutes of their formation (Cheung and Cousin, 2012; Glyvuk et al., 2010; Kononenko et al., 2014; Stevens et al., 2012; Wu et al., 2014c). Using an approach employed previously (Stevens et al., 2012), we treated the boutons with high K^+^ followed by an incubation in 5 mM K^+^ and 0 mM Ca^2+^ solution for 10 or 20 min, allowing the SVs to reform from the bulk endosomes. In controls (*nSyb-GAL4)*, the number and area of the induced bulk endosomes reverted to almost basal levels within 10 min (Figure 2c–d; Figure 2—figure supplement 2), demonstrating proper SV reformation. Interestingly, upon expression of low levels of PLC_δ1_-PH-EGFP, the induced bulk endosomes remained after 10 min and 20 min recovery periods, yet ADBE was only partially impaired (Figure 2c–d; Figure 2—figure supplement 2). In contrast, SV reformation actively occurred in the boutons expressing high levels of PLC_δ1_-PHS39R-EGFP (Figure 2c–d; Figure 2—figure supplement 2). Furthermore, we observed a loss of SV reformation ability upon mild overexpression of Synj (Figure 2c–d; Figure 2—figure supplement 2). Hence, the formation of PI(4,5)P_2_ microdomains is essential for both ADBE and SV reformation from the bulk endosome, with the latter event being elicited by relatively high levels of PI(4,5)P_2_. Note that perturbation of PI(4,5)P_2_ microdomain formation did not cause SV accumulation on the bulk endosome during recovery periods, indicating that PI(4,5)P_2_ microdomains may play an early role in SV reformation from bulk endosomes.

### PI(4,5)P_2_ microdomains are established via a positive feedback loop of fwe and PI(4,5)P_2_

**Figure 3.**
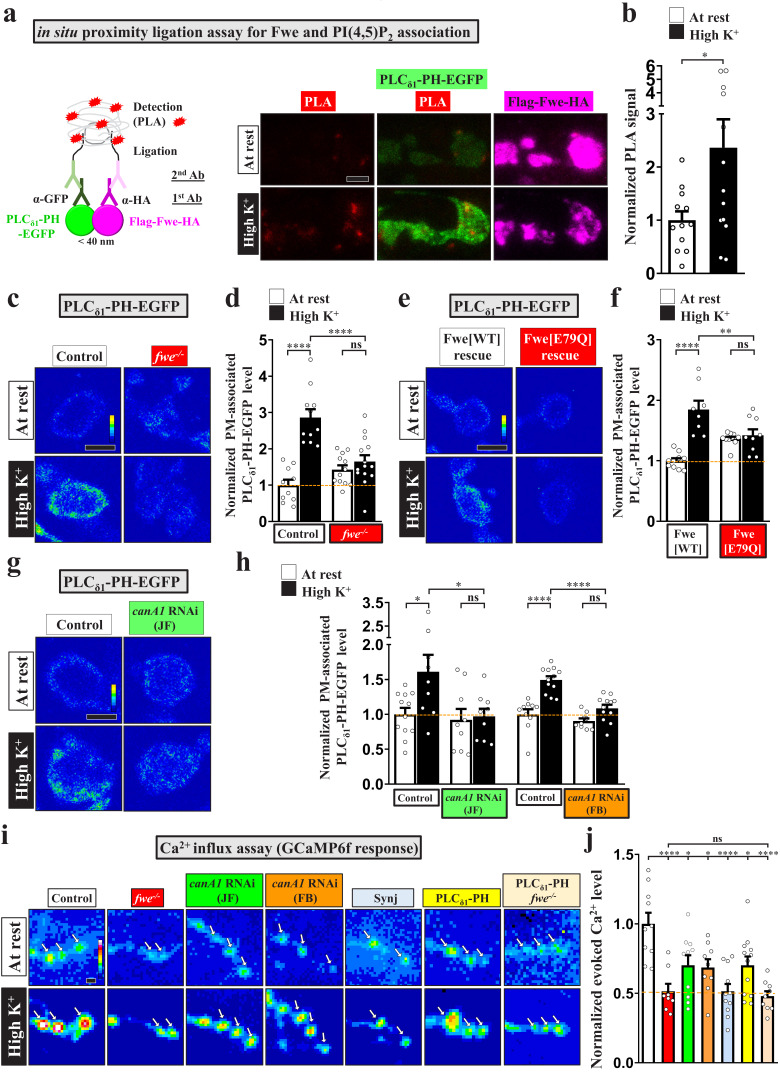
Fwe and PI(4,5)P_2_ form a positive feedback loop to establish PI(4,5)P_2_ microdomains. (**a–b**) Fwe and PLC_δ1_-PH-EGFP interact upon intense stimulation. (**a**) (Left) A schematic for PLA. (Right) Z-projected confocal images of *fwe* mutant boutons co-expressing Flag-Fwe-HA and PLC_δ1_-PH-EGFP (*nSyb-GAL4/UAS-Flag-fwe-HA*/*UAS-PLC_δ1_-PH-EGFP in fwe^DB25/DB56^* at 25°C). After resting condition or high K^+^ stimulation, the boutons were subjected to PLA. α-GFP and α-HA stained for PLC_δ1_-PH-EGFP (green) and Flag-Fwe-HA (magenta), respectively. PLA signals (red). (**b**) Quantification data for PLA signal intensities, normalized to the value of the resting condition. (**c–f**) Loss of Fwe or its Ca^2+^ channel activity perturbs PI(4,5)P_2_ microdomain formation. (**c and e**). (**c**) Single-plane confocal images of the boutons neuronally expressing *UAS-PLC_δ1_-PH-EGFP* in *fwe^DB25/+^* (control) or *fwe^DB25/DB56^* at 25°C. The boutons were subjected to resting condition or high K^+^ stimulation, followed by α-GFP immunostaining. Quantification data for PLC_δ1_-PH-EGFP staining intensity are shown, normalized to the value of the resting condition of controls (**d**). (**e**) Single-plane confocal images of the boutons neuronally expressing *lexAop2-PLC_δ1_-PH-EGFP* using *vglut-lexA* in wild-type Fwe rescue boutons (*vglut-lexA/lexAop2-PLC_δ1_-PH-EGFP, nSyb(w)-GAL4/UAS-flag-fwe-RB-HA* in *fwe^DB25/fweDB56^* at 25°C), or FweE79Q rescue boutons (*vglut-lexA/lexAop2-PLC_δ1_-PH-EGFP, nSyb(w)-GAL4/UAS-flag-fweE79Q-RB-HA* in *fwe^DB25/fweDB5^* at 25°C). When the weak *nSyb(w)-GAL4* driver drove low expression of *UAS-fwe* transgenes, this binary system (*vglut-lexA/lexAop2-PLC_δ1_-PH-EGFP*) was used to produce detectable levels of PLC_δ1_-PH-EGFP in boutons. Quantification data for PLC_δ1_-PH-EGFP staining intensity are shown, normalized to the value of the resting condition of Fwe-rescued boutons (**f**). (**g–h**) *canA1* RNAi knockdown impairs PI(4,5)P_2_ microdomain formation. Single-plane confocal images of the boutons co-expressing *UAS-PLC_δ1_-PH-EGFP* with *UAS-RFP* (control), *UAS-canA1-RNAi (TRiP.JF01871),* or *UAS-canA1-RNAi (FB4)* using *nSyb-GAL4*. The larvae were reared at 25°C. After resting condition or high K^+^ stimulation, the boutons were stained with α-GFP. (**h**) Quantification data for PLC_δ1_-PH-EGFP staining intensities, normalized to the value of the resting condition of controls. (**i–j**) Blockade of the PI(4,5)P_2_ microdomains attenuates Fwe Ca^2+^ conductance. (**i**) Snapshot Ca^2+^ images of the boutons (arrows) expressing *lexAop2-GCaMP6f* using *vglut-lexA*. The larvae of control (*w^1118^*), *fwe* mutant (*fwe^DB25/DB56^*), *canA1* RNAi (JF) (*nSyb-GAL4/UAS-canA1-RNAi (TRiP.JF01871)*), *canA1* RNAi (FB) (*nSyb-GAL4/UAS-canA1-RNAi (FB4)*), Synj overexpression (*nSyb-GAL4/UAS-synj*), PLC_δ1_-PH-APEX2-HA expression (*vglut-lexA/LexAop2-PLC_δ1_-PH-APEX2-HA*), or *fwe* mutant expressing PLC_δ1_-PH-APEX2-HA (*vglut-lexA/LexAop2-PLC_δ1_-PH-APEX2-HA* in *fwe^DB25/DB56^*) were reared at 25°C. Imaging was taken in the fifth minute for one minute after high K^+^ (2 mM Ca^2+^) stimulation. (**j**) Quantification data for evoked Ca^2+^ level, normalized to the value of controls. Evoked Ca^2+^ levels are shown as the increase in GCaMP6f fluorescence under high K^+^ stimulation. Individual data values are shown in graphs. p values: ns, not significant; *p<0.05; **p<0.01; ****p<0.0001. Mean ± SEM. Scale bar: 2 µm. Statistics: Student *t*-test (**b**). One-way ANOVA with Tukey’s post hoc test (**d, f, h, j**). Figure 3—source data 1.Source data for Figure 3.

We have previously shown that the SV-associated Ca^2+^ channel Flower (Fwe) elevates presynaptic Ca^2+^ levels in response to strong stimuli to trigger ADBE (Yao et al., 2009; Yao et al., 2017). Given the Ca^2+^ dependence of PI(4,5)P_2_ microdomains (Figure 1d), we hypothesized that exocytosis evoked by intense stimulation promotes Fwe clustering at periactive zones, where it may provide the Ca^2+^ influx to induce PI(4,5)P_2_ microdomain formation. To test this hypothesis, we first conducted a proximity ligation assay (PLA) (Söderberg et al., 2008) to investigate if there is a close association between Fwe and PI(4,5)P_2_ in response to stimulation. In our PLA (Figure 3a), *UAS-Flag-Fwe-HA* was expressed in a *fwe* mutant background to replace endogenous Fwe protein with a tagged protein, and expression of *UAS-PLC_δ1_-PH-EGFP* reported the localization of PI(4,5)P_2_. Primary antibodies against the HA tag and EGFP protein were used to detect interactions between Flag-Fwe-HA and PLC_δ1_-PH-EGFP. The PLA signal was low in resting boutons, whereas high K^+^ treatment significantly increased PLA signal intensity (Figure 3a–b). Therefore, these results suggest that Fwe and PI(4,5)P_2_ are closely colocalized when intense stimulation triggers PI(4,5)P_2_ microdomain formation.

Next, we investigated the effect of loss of Fwe on PI(4,5)P_2_ microdomain formation. Basal levels of PI(4,5)P_2_ were not affected in the *fwe* mutant relative to control (Figure 3c–d). However, intense stimulation failed to elicit PI(4,5)P_2_ microdomain formation in the *fwe* mutant (Figure 3c–d), revealing a crucial role for Fwe in this process. To determine if the Ca^2+^ channel activity of Fwe is responsible for this activity, we conducted rescue experiments based on our previous report in which *fwe* mutant boutons exhibited expression of the wild-type Fwe transgene or the FweE79Q mutant transgene that has reduced Ca*^2+^* conductance (Yao et al., 2017). We found that the wild-type Fwe transgene promoted PI(4,5)P_2_ microdomain formation, whereas the FweE79Q mutant transgene lost that ability (Figure 3e–f). Thus, Fwe triggers the formation of PI(4,5)P_2_ microdomains in a Ca^2+^ channel-dependent manner.

Calmodulin and Calcineurin are thought to be the Ca^2+^ sensors for ADBE (Evans and Cousin, 2007; Jin et al., 2019; Marks and McMahon, 1998; Sun et al., 2010; Wu et al., 2009; Wu et al., 2014b). The Calcineurin complex dephosphorylates PI(4,5)P_2_ metabolic enzymes, including Synj and Phosphatidylinositol 4-phosphate 5-kinase Iγ (PIPKIγ) (Cousin and Robinson, 2001; Lee et al., 2005; Lee et al., 2004; van den Bout and Divecha, 2009). *Drosophila* possesses three isoforms of the catalytic subunit of Calcineurin, i.e., CanA1, CanA-14F, and Pp2B-14D. CanA1 regulates development of *Drosophila* NMJ boutons (Wong et al., 2014). Therefore, we knocked down *canA1* in neurons by using two independent *UAS-canA1-RNAi* constructs, *UAS-canA1-RNAi(JF01871)* (Wong et al., 2014) and *UAS-canA1-RNAi(FB4)* (Dijkers and O'Farrell, 2007). Similar to the effect of loss of Fwe, reducing CanA1 levels via expression of either RNAi construct greatly suppressed the formation of PI(4,5)P_2_ microdomains compared to the control (Figure 3g–h), indicating that Calcineurin mediates the Ca^2+^ influx conducted by Fwe to induce PI(4,5)P_2_ microdomains.

Our previous Ca^2+^ imaging data showed that intense stimulation activates Fwe to increase presynaptic Ca^2+^ concentrations (Yao et al., 2017). We measured intracellular Ca^2+^ levels with the Ca^2+^ indicator GCaMP6f (Chen et al., 2013), and found that whereas control boutons exhibited a robust increase in intracellular Ca^2+^ upon high K^+^ stimulation, *fwe* mutant boutons exhibit an impaired Ca^2+^ response (Figure 3i–j). Unexpectedly, *canA1* knockdown also elicited the same deficient Ca^2+^ response (Figure 3i–j). Similar suppressive effects were obtained upon overexpressing Synj or the PLC_δ1_-PH domain (Figure 3i–j). Hence, Calcineurin activation may increase PI(4,5)P_2_ activity, which may in turn promote the Ca^2+^ channel activity of Fwe to further increase intracellular Ca^2+^ levels. To investigate the potential for such feedback regulation, we expressed the PLC_δ1_-PH domain in a loss of Fwe background. Expression of the PLC_δ1_-PH domain did not rescue the low Ca^2+^ concentration caused by the *fwe* mutation (Figure 3i–j), showing that the Ca^2+^ suppression exerted by the PLC_δ1_-PH domain indeed depends on Fwe. These findings support that a positive feedback loop involving Fwe and PI(4,5)P_2_ is responsible for the formation of PI(4,5)P_2_ microdomains.

### PI(4,5)P_2_ gates fwe

**Figure 4.**
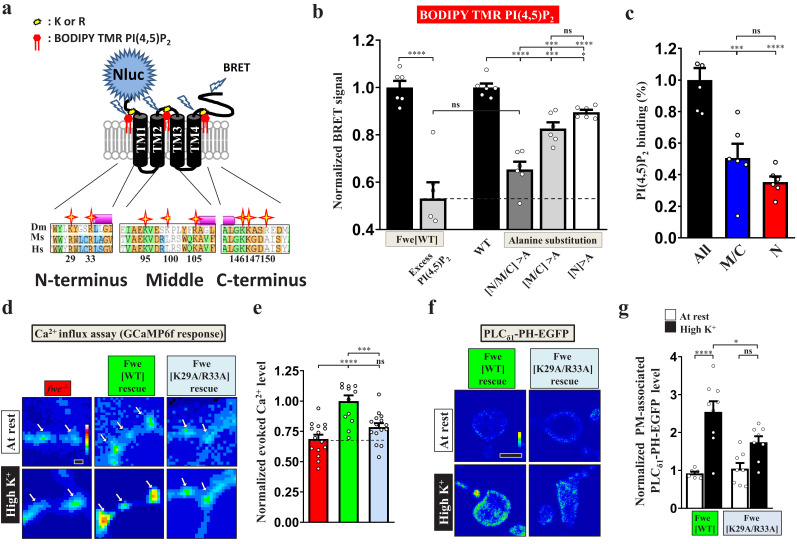
PI(4,5)P_2_ binds to Fwe and promotes its Ca^2+^ channelling activity. (**a–c**) PI(4,5)P_2_ binds Fwe. (**a**) A schematic of the Fwe structure and a BRET assay. Stars highlight conserved lysine (**K**) and arginine (**R**) in juxta-transmembrane regions: N-terminus (**N**), middle-domain (**M**), and C-terminus (**C**). *Drosophila melanogaster* (Dm). *Mus Musculus* (Ms). *Human Sapiens* (Hs). N-terminal fusion of Nluc to Fwe allows detection of PI(4,5)P_2_ binding. A 1D4 epitope was used for protein purification. (**b**) Nluc-Fwe-1D4 in micelles excites BODIPY-TMR-conjugated PI(4,5)P_2_ to emit BRET signal, which is decreased by competitive cold PI(4,5)P_2_ (1 mM). Alanine substitution of positively-charged residues in all regions (N/M/C > A), both middle-domain and C-terminus (M/C > A), or N-terminus (N > A) reduced BRET signals. (**c**) Corresponding PI(4,5)P_2_ binding ability was calculated by subtracting the signal values of N/M/C > A, M/C > A or N > A from that for WT. Quantification data was normalized to the signal value of all mutations (N/M/C). (**d**) PI(4,5)P_2_ controls Fwe channel activity. Snapshot Ca^2+^ images of the boutons (arrows) expressing *lexAop2-GCaMP6f* using *vglut-lexA. fwe* mutant (*fwe^DB25/DB56^*). HA-Fwe[WT]-APEX2 rescue (*nSyb-GAL4/UAS-HA-Fwe[WT]-APEX2 in fwe^DB25/DB56^*). HA-Fwe[K29A/R33A]-APEX2 rescue (*nSyb-GAL4/UAS-HA-Fwe[K29A/R33A]-APEX2 in fwe^DB25/DB56^*). The larvae were reared at 25°C. Images were taken in the fifth minute for one minute after high K^+^ stimulation. (**e**) Quantification data for evoked Ca^2+^ level, normalized to the value of HA-Fwe-APEX2 rescue larvae. (**f**) Single-plane confocal images of the boutons expressing *UAS-PLC_δ1_-PH-EGFP*. The larvae were reared at 25°C. After resting conditions or high K^+^ stimulation, the boutons were stained with α-GFP. (**g**) Quantification data for the PLC_δ1_-PH-EGFP staining intensity, normalized to the value of the resting condition of HA-Fwe-APEX2 rescue larvae. Individual data values are shown in graphs. p values: ns, not significant; *p<0.05; ***p<0.001; ****p<0.0001. Mean ± SEM. Scale bar: 2 µm (**d, f**). Statistics: Student *t*-test (**b**). One-way ANOVA with Tukey’s post hoc test (**b, c, e, g**). Figure 4—source data 1.Source data for Figure 4.

A well-known function of PI(4,5)P_2_ is to modulate ion channel activity through its electrostatic binding to clustered positively-charged amino acids adjacent to the transmembrane domains of ion channels (Hille et al., 2015; Suh and Hille, 2008). Through protein alignment analysis, we found tandem positively-charged amino acids, including lysine (K) and arginine (R), in the intracellular juxta-transmembrane regions of Fwe. These residues are evolutionarily conserved in mice and humans (Figure 4a), whereas other cytosolic residues show poor conservation. This feature inspired us to test the potential impact of PI(4,5)P_2_ on the channel function of Fwe. To determine direct interaction between Fwe and PI(4,5)P_2_, we conducted a nanoluciferase (Nluc)-based bioluminance resonance energy transfer (BRET) assay (Cabanos et al., 2017). In our BRET assay (Figure 4a), upon ion channel binding of BODIPY-TMR-conjugated PI(4,5)P_2_, illumination of Nluc-fused ion channels wrapped in detergent-formed micelles can excite BODIPY-TMR-conjugated PI(4,5)P_2_ to emit a BRET signal. As shown in Figure 4b, we reconstituted the micelles containing purified Nluc-Fwe-1D4 fusion proteins and, after adding BODIPY-TMR-PI(4,5)P_2_ and furimazine (a Nluc substrate), we observed a remarkable increase in BRET signal. Excess cold PI(4,5)P_2_ reduced the signal to ~50% of the BRET signals by competing for the PI(4,5)P_2_ binding sites in Fwe, suggesting direct PI(4,5)P_2_ binding to Fwe. To assess the involvement of the positively-charged amino acids of Fwe in PI(4,5)P_2_ binding, we mutated all of the clustered positively-charged amino acids to non-charged alanine to eliminate the electrostatic interactions. Residue substitution resulted in a significant reduction in BRET signal, comparable to the competitive effect attributable to provision of excess cold PI(4,5)P_2_. Therefore, the majority of the PI(4,5)P_2_ binding activity of Fwe is mediated by these positively-charged amino acids. Specifically, alanine substitution of residues in both the middle (K95, K100, R105) and C-terminal (K146, K147, R150) regions of Fwe reduced PI(4,5)P_2_-specific binding (Figure 4b–c). Moreover, when N-terminal residues (K29, R33) of Fwe were further substituted with alanines, binding of PI(4,5)P_2_ was also reduced (Figure 4b–c). Hence, these in vitro assays reveal that Fwe directly binds PI(4,5)P_2_ through multiple regions.

**Figure 4—figure supplement 1.**
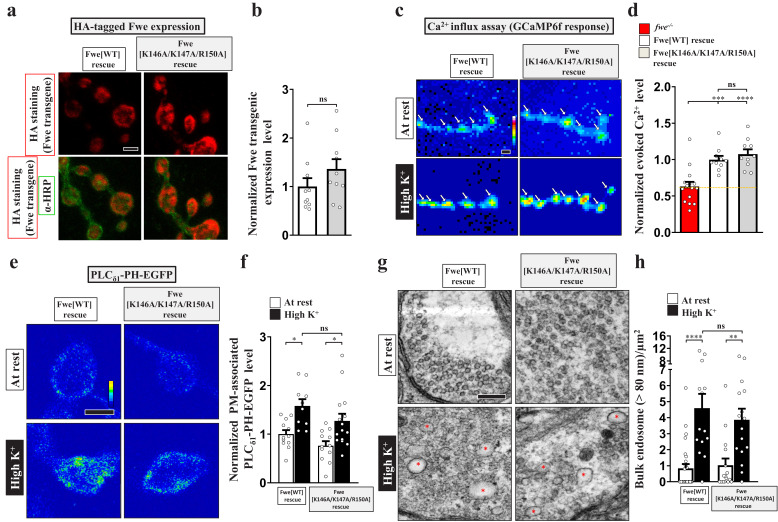
The C-terminal residues (K146/K147/R150) of Fwe are not involved in regulating Ca^2+^ channel activity. (**a–b**) Alanine substitution of the C-terminal residues K146/K147/R150 does not influence the expression or SV localization of Fwe. (**a**) Z-projected confocal images of NMJ boutons derived from the larvae of Flag-Fwe[WT]-HA rescue (*nSyb-GAL4/UAS-Flag-Fwe-HA in fwe^DB25/DB56^*) or Flag-Fwe[K146A/K147A/R150A]-HA rescue *(nSyb-GAL4/UAS-Flag-Fwe[K146A/K147A/R150A]-HA in fwe^DB25/DB56^*) lines. The larvae were reared at 25°C. α-HA (red) and α-HRP (green) staining was used to detect Fwe transgenic proteins and neuronal membranes, respectively. α-HRP staining was also used as the staining control. (**b**) Quantification data for the staining intensity ratio of α-HA to α-HRP, normalized to the value of the Flag-Fwe[WT]-HA rescue line. (**c–h**) Alanine substitution of the C-terminal residues K146/K147/R150 does not influence Ca^2+^ channel activity and the ability to induce PI(4,5)P_2_ microdomains and ADBE. (**c**) Snapshot Ca^2+^ images of the NMJ boutons. *fwe^-/-^* (*fwe^DB25/DB56^*). The boutons were subjected to 5 min stimulation of 90 mM K^+^/2 mM Ca^2+^. The images were taken in the fifth minute. Evoked Ca^2+^ levels are represented as the increase in GCaMP6f fluorescence in response to high K^+^ stimulation. (**d**) Quantification data for evoked Ca^2+^ level, normalized with the value of the Flag-Fwe[WT]-HA rescue line. (**e**) Single-plane confocal images of the NMJ boutons. The boutons were subjected to the resting condition (10 min incubation of 5 mM K^+^/0 mM Ca^2+^) or high K^+^ stimulation (10 min stimulation of 90 mM K^+^/2 mM Ca^2+^), followed by chemical fixation and α-GFP immunostaining. (**f**) Quantification data for the PLC_δ1_-PH-EGFP staining intensity, normalized to the value of the resting condition of the Flag-Fwe[WT]-HA rescue line. (**g**) TEM images of NMJ boutons. TEM processing was performed under the following conditions: at rest (10 min incubation of 5 mM K^+^/0 mM Ca^2+^); or high K^+^ (10 min stimulation of 90 mM K^+^/2 mM Ca^2+^). Bulk endosomes (red asterisks). (**h**) Quantification data of total numbers of bulk endosomes per bouton area. Individual data values are shown in the graphs. p values: ns, not significant; *p<0.05; **p<0.01; ***p<0.001; ****p<0.0001. Mean ± SEM. Scale bar: 2 µm (**a, c, e**), 500 nm (**g**). Statistics: Student *t*-test (**b**). One-way ANOVA with Tukey’s post hoc test (**d, f, h**). Figure 4—figure supplement 1—source data 1.Source data for Figure 4—figure supplement 1.

**Figure 4—figure supplement 2.**
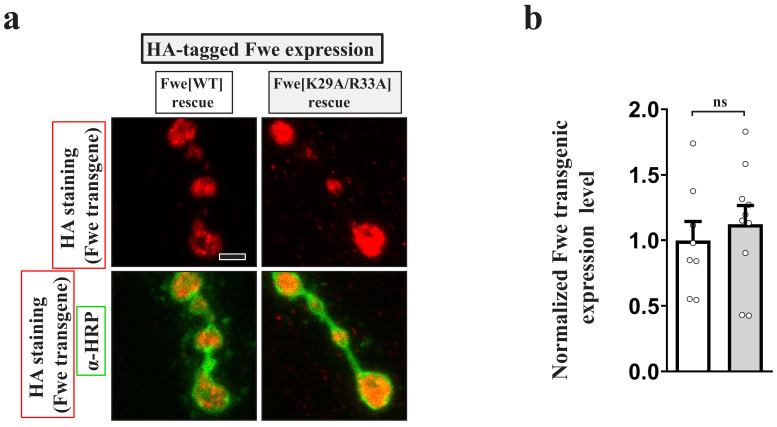
Expression of Fwe[R29A/K33A] in boutons. (**a–b**) The Fwe[K29A/R33A] mutant variant properly localizes to the presynaptic compartment. (**a**) Z-projected confocal images of NMJ boutons derived from the larvae of the HA-Fwe[WT]-APEX2 rescue (*nSyb-GAL4/UAS-HA-Fwe[WT]-APEX2 in fwe^DB25/DB56^*) or HA-Fwe[K29A/R33A]-APEX2 rescue *(nSyb-GAL4/UAS- HA-Fwe[K29A/R33A]-APEX2 in fwe^DB25/DB56^*) lines. The larave were reared at 25°C. α-HA (red) and α-HRP (green) staining was used to detect Fwe transgenic proteins and neuronal membranes, respectively. α-HRP staining was also used as the staining control. (**b**) Quantification data for the staining intensity ratio of α-HA to α-HRP, normalized to the value of the Flag-Fwe[WT]-HA rescue line. Individual data values are shown in the graphs. p values: ns, not significant. Scale bar: 2 µm. Statistics: Student *t*-test. Figure 4—figure supplement 2—source data 1.Source data for Figure 4—figure supplement 2.

To directly test how PI(4,5)P_2_ affects Flower Ca^2+^ channel function, we generated *UAS* transgenes for the Fwe variants in which all or subsets of positively-charged amino acids were mutated to alanine and performed a mutant rescue experiment using *nSyb-GAL4*. Mutations of all nine residues or only those in the middle region (K95/K100/R105) led to very low protein expression levels, preventing further study. However, alanine substitution of C-terminal residues K146/K147/R150 did not affect SV localization of Fwe or its ability to regulate presynaptic Ca^2+^ concentration and induce PI(4,5)P_2_ microdomain formation (Figure 4—figure supplement 1a–f), suggesting that these residues do not play a regulatory role in Fwe channel activity. We also mutated the N-terminal residues K29/R33. The resulting K29A/R33A variant was still able to properly localize to presynaptic terminals (Figure 4—figure supplement 2a–b). However, upon high K^+^ stimulation, the K29A/R33A variant lost that ability to maintain proper intracellular Ca^2+^ levels (Figure 4d–e). Moreover, that variant failed to promote PI(4,5)P_2_ microdomain formation upon high K^+^ stimulation (Figure 4f–g). These results reveal that the positive feedback loop involving Fwe and PI(4,5)P_2_ relies on PI(4,5)P_2_-dependent gating control of Fwe.

### Blockade of the positive feedback loop reduces ADBE and SV reformation from bulk endosomes

**Figure 5.**
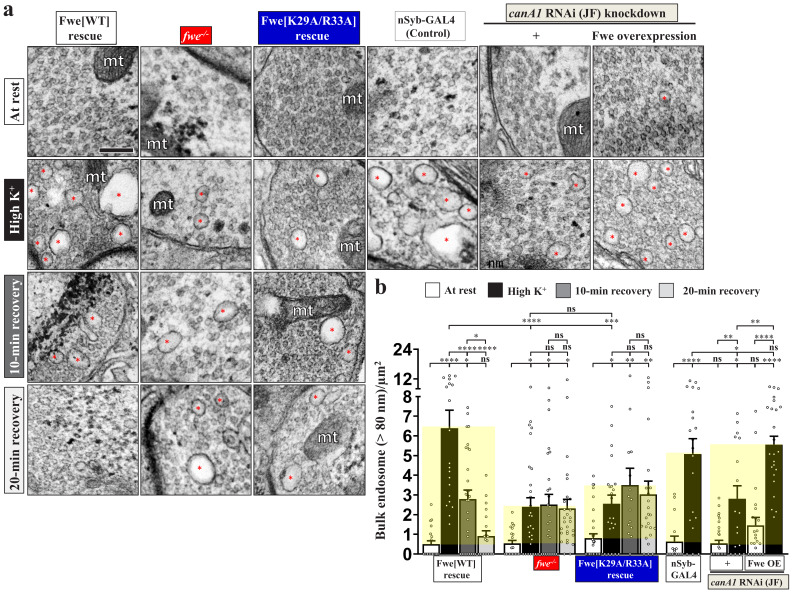
Perturbations of the positive feedback loop involving Fwe and PI(4,5)P_2_ suppress ADBE and SV reformation from bulk endosomes. (**a**) TEM images of the boutons of HA-Fwe-APEX2 rescue (*nSyb-GAL4/UAS-HA-Fwe-APEX2 in fwe^DB25/DB56^* at 25°C), *fwe* mutant (*fwe^DB25/DB56^* at 25°C), HA-Fwe[K29A/R33A]-APEX2 rescue (*nSyb-GAL4/UAS-HA-Fwe[K29A/R33A]-APEX2 in fwe^DB25/DB56^* at 25°C), control (*nSyb-GAL4* at 25°C), *canA1* RNAi (JF) knockdown (*nSyb-GAL4/UAS-canA1-RNAi (TRiP.JF01871)* at 29°C), *canA1* RNAi (JF) knockdown plus Fwe overexpression (*nSyb-GAL4/UAS-canA1-RNAi (TRiP.JF01871)/UAS-HA-Fwe-APEX2* at 29°C). TEM processing was performed after the following treatments: at rest (10 min incubation of 5 mM K^+^/0 mM Ca^2+^); high K^+^ stimulation (10 min stimulation of 90 mM K^+^/2 mM Ca^2+^); 10 min recovery (10 min stimulation of 90 mM K^+^/2 mM Ca^2+^, followed by 10 min incubation of 5 mM K^+^/0 mM Ca^2+^); or 20 min recovery (10 min stimulation of 90 mM K^+^/2 mM Ca^2+^, followed by 20 min incubation of 5 mM K^+^/0 mM Ca^2+^). Bulk endosomes (>80 nm in diameter, red asterisks). Mitochondria (mt). (**b**) Quantification data of total numbers of bulk endosomes per bouton area. Individual data values are shown in graphs. p values: ns, not significant; *p<0.05; **p<0.01; ***p<0.001; ****p<0.0001. Mean ± SEM. Scale bar: 500 nm. Statistics: one-way ANOVA with Tukey’s post hoc test. Figure 5—source data 1.Source data for Figure 5.

Next, we assessed the impact of the Fwe and PI(4,5)P_2_ regulatory feedback loop on ADBE. Loss of Fwe severely impaired formation of bulk endosomes induced by ADBE under high K^+^ conditions compared to the *fwe* mutant rescue control (Figure 5a–b), consistent with our previous findings (Yao et al., 2017). Next, we found that expression of wild-type Fwe protein or the K146A/K147A/R150A mutant variant restored proper ADBE in the *fwe* mutant background (Figure 5a–b; Figure 4—figure supplement 1g–h), whereas expression of the K29A/R33A variant failed to rescue ADBE deficiency (Figure 5a–b). Consistent with the suppressive effects caused by expression of the PLCδ1-PH domain or Synj (Figure 2), all of the bulk endosomes that remained in boutons lacking *fwe* could not generate new SVs during a 10 min or even 20 min recovery period (Figure 5a–b; Figure 5—figure supplement 1). Expression of wild-type Fwe protein but not the K29A/R33A mutant variant rescued this defect (Figure 5a–b; Figure 5—figure supplement 1).

**Figure 5—figure supplement 1.**
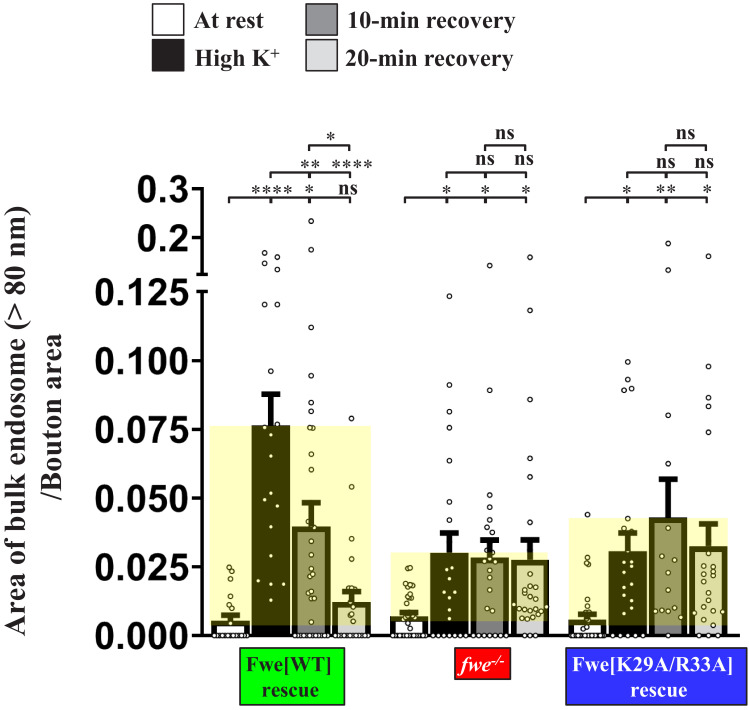
Blockade of the positive feedback loop between Fwe and PI(4,5)P_2_ abolishes SV reformation from the bulk endosome. Quantification data for the ratio of total area of bulk endosomes to bouton area of the indicated genotypes. Individual data values are shown in the graphs. p values: ns, not significant; *p<0.05; **p<0.001; ****p<0.0001. Mean ± SEM. Statistics: One-way ANOVA with Tukey’s post hoc test. Figure 5—figure supplement 1—source data 1.Source data for Figure 5—figure supplement 1.

Neuronal *canA1* knockdown by expressing *canA1* RNAi construct also impaired ADBE relative to the *nSyb-GAL4* control (Figure 5a–b). To verify if CanA1 regulates ADBE in an Fwe-dependent manner, we overexpressed Fwe to increase the intracellular Ca^2+^ levels under *canA1 RNAi* knockdown conditions. Fwe overexpression significantly reversed the ADBE defect (Figure 5a–b), suggesting that increasing Fwe-dependent Ca^2+^ influx can augment activation of the residual CanA1 enzymes and thus normalize downstream ADBE. Taken together with our results reported in previous sections, we propose that the positive feedback loop involving Fwe, CanA1 and PI(4,5)P_2_ compartmentalizes PI(4,5)P_2_ microdomains at the periactive zone of boutons to dictate and coordinate ADBE and subsequent SV reformation.

### PI(4,5)P_2_ facilitates retrieval of fwe to bulk endosomes

**Figure 6.**
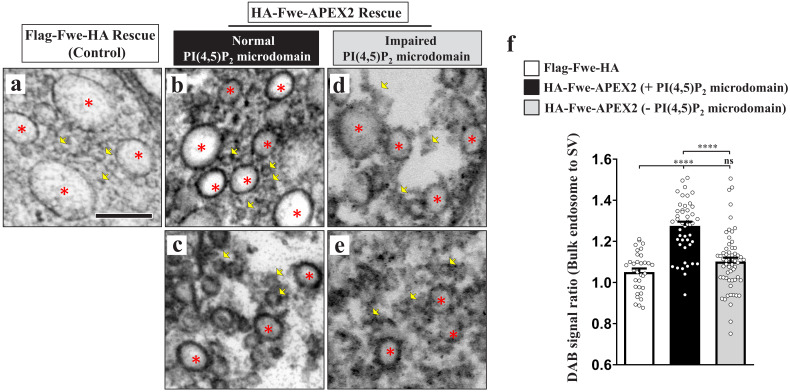
PI(4,5)P_2_ facilitates recycling of Fwe to bulk endosomes. (**a–f**) PI(4,5)P_2_ retrieves Fwe to bulk endosomes. (**a–e**) TEM images of the boutons of Flag-Fwe-HA rescue (*nSyb-GAL4/UAS-Flag-Fwe-HA in fwe^DB25/DB56^* at 25°C, **a**), HA-Fwe-APEX2 rescue (*nSyb-GAL4/UAS-HA-Fwe-APEX2 in fwe^DB25/DB56^* at 25°C, **b–c**), or HA-Fwe-APEX2 rescue coexpressing Synj (*nSyb-GAL4/UAS-HA-Fwe-APEX2*/*UAS-synj in fwe^DB25/DB56^* at 25°C, **d–e**). After high K^+^ stimulation, the boutons were subjected to DAB labeling. (**a**) The Flag-Fwe-HA rescue boutons presented DAB-negative bulk endosomes (red asterisks) and SVs (yellow arrows). (**b and c**) In the HA-Fwe-APEX2 rescue boutons, DAB signals on bulk endosomes (red asterisks) were higher than those on the SVs (yellow arrows). The views in b and c are from different boutons. (**d and e**) Under the condition of Synj overexpression, perturbation of PI(4,5)P_2_ microdomain formation predominantly reduced DAB levels on the bulk endosomes (red asterisks). Views in d and e are from different boutons. (**f**) Quantification data for the DAB staining intensity ratio of bulk endosomes to surrounding SVs. The number of bulk endosomes, NMJ boutons, and larvae counted (Flag-Fwe-HA rescue control): Bulk endosomes (n = 33) derived from 5 NMJ boutons of two different larvae; HA-Fwe-APEX2 rescue: Bulk endosomes (n = 47) derived from 6 NMJ boutons of two different larvae; HA-Fwe-APEX2 rescue expressing Synj: Bulk endosomes (n = 59) derived from 9 NMJ boutons of two different larvae. Individual values were shown in graphs. p values: ns, not significant; ****p<0.0001. Mean ± SEM. Scale bar: 200 nm (**a–e**). Statistics: one-way ANOVA with Tukey’s post hoc test. Figure 6—source data 1.Source data for Figure 6.

It was reported recently that a SV protein sorting process occurs during ADBE (Kokotos et al., 2018; Nicholson-Fish et al., 2015). VAMP4, a v-SNARE protein, is essential for ADBE to proceed, and it is selectively retrieved by ADBE (Kokotos et al., 2018; Nicholson-Fish et al., 2015). Given the important role of Fwe in triggering ADBE, we wondered if Fwe is sorted to bulk endosomes during ADBE. To visualize the vesicular localization of Fwe, we rescued the *fwe* mutant by expressing a *UAS* transgene of the APEX2 fusion protein of Fwe (*UAS-HA-Fwe-APEX2*), and then conducted diaminobenzidine (DAB) labeling and TEM. By means of confocal microscopy, we observed that HA-Fwe-APEX2 immunostaining signals were properly present on the SVs marked by Syt and Csp immunostaining (Figure 6—figure supplement 1a). Furthermore, expression of this fusion protein was able to rescue the endocytic defects (Figures 4 and 5) and early animal lethality (not shown) caused by loss of *fwe*. Therefore, HA-Fwe-APEX2 is functionally equivalent to endogenous Fwe. APEX2 is an engineered peroxidase that is capable of catalyzing DAB polymerization and proximal deposition, with the DAB polymers binding electron-dense osmium to enhance electron microscopy contrast (Lam et al., 2015). Whereas no DAB staining signals were observed in SVs in the Flag-Fwe-HA rescue control boutons stimulated by high K^+^ (yellow arrows, Figure 6—figure supplement 1b'; Figure 6a), the SV-based localization of HA-Fwe-APEX2 was clearly revealed by DAB staining in HA-Fwe-APEX2-rescued boutons under the resting and stimulation conditions (yellow arrows, Figure 6—figure supplement 1c–d). Moreover, specific DAB labeling also revealed the presynaptic plasma membrane localization of HA-Fwe-APEX2 (white arrows, Figure 6—figure supplement 1b–c), an outcome consistent with previous immunogold staining assays on endogenous Fwe (Yao et al., 2009). Upon high K^+^ stimulation, SV and plasma membrane localizations were retained (Figure 6—figure supplement 1d'). Remarkably, DAB signals were apparent on all bulk endosomes (Figure 6b–c; Figure 6—figure supplement 1d and d'). To minimize staining variability across boutons, we compared DAB staining intensities on bulk endosomes and SVs in the same boutons. Compared to Flag-Fwe-HA rescue boutons, in HA-Fwe-APEX2 rescue boutons, the staining intensities of bulk endosomes were more abundant compared to those of the surrounding SVs (Figure 6f), revealing a mechanism by which Fwe is recycled to bulk endosomes after it initiates ADBE.

**Figure 6—figure supplement 1.**
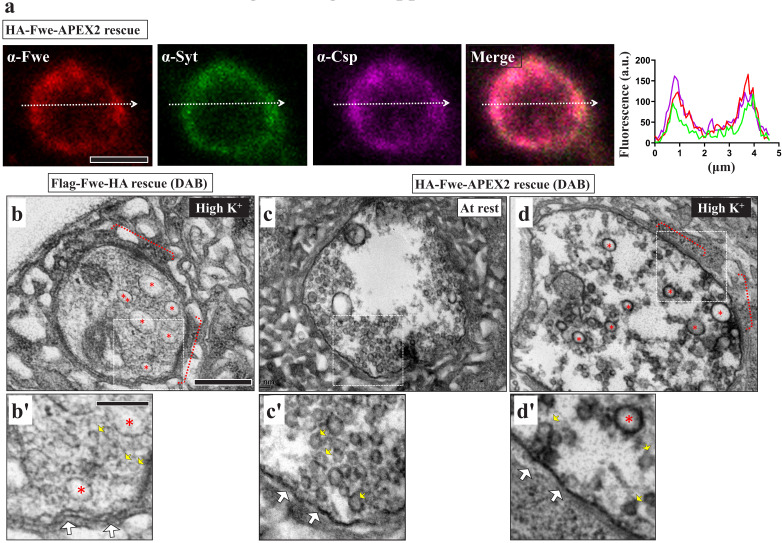
HA-Fwe-APEX2 behaves like endogenous Fwe. (**a**) HA-Fwe-APEX2 localizes in the SVs. Single-plane confocal images of NMJ boutons of the HA-Fwe-APEX2 rescue line (*nSyb-GAL4/UAS-HA-Fwe[WT]-APEX2 in fwe^DB25/DB56^*). The boutons were stained for Fwe (red) and the SV markers, Syt (green), and Csp (magenta). Line plot profiles of absolute fluorescence units (a.u.) of immunostaining signals are shown at right. (**b–d**) TEM images of the boutons of Flag-Fwe-HA rescue (*nSyb-GAL4/UAS-Flag-Fwe-HA in fwe^DB25/DB56^* at 25°C), (**b**) or HA-Fwe-APEX2 rescue (*nSyb-GAL4/UAS-HA-Fwe-APEX2*) *in fwe^DB25/DB56^* at 25°C, (**c and d**) lines. The boutons were subjected to the resting condition (**c**) or high K^+^ stimulation (**b and d**), followed by DAB labeling. High-magnification images are shown at bottom. In the Flag-Fwe-HA rescue boutons, SVs (yellow arrows), bulk endosomes (red asterisks), and plasma membrane (white arrows) lack specific DAB signals. In the HA-Fwe-APEX2 rescue boutons at rest, both SVs (yellow arrows) and plasma membrane (white arrows) were significantly labelled with DAB. Upon high stimulation, high levels of DAB signals were observed on the bulk endosomes of HA-Fwe-APEX2 rescue boutons. Active zones (red dashed lines). Scale bar: 2 µm (**a**), 500 nm (**b, c, d**), 200 nm (**b’, c’, d’**).

PI(4,5)P_2_ is known to recruit adaptor protein complexes to sort SV proteins to the nascent SV during CME (Saheki and De Camilli, 2012). Next, we assessed if PI(4,5)P_2_ microdomain formation may be involved in sorting of Fwe to bulk endosomes. When PI(4,5)P_2_ microdomains were perturbed by Synj overexpression (Figure 2b), the bulk endosome localization of HA-Fwe-APEX2 was significantly reduced (Figure 6d–f), whereas there was only a mild reduction in its SV localization (Figure 6d–e). Therefore, in addition to initiating ADBE, PI(4,5)P_2_ microdomains play a role in facilitating the retrieval of Fwe to the bulk endosome, enabling ADBE to remove its trigger via a negative feedback regulatory mechanism and reducing endocytosis to prevent excess membrane uptake.

## Discussion

**Figure 7.**
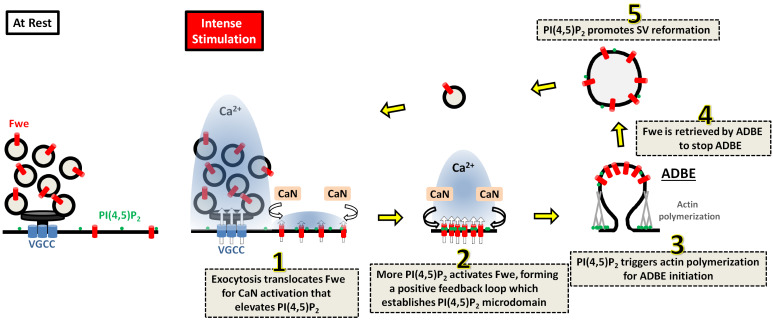
A proposed model for the role of Fwe-dependent PI(4,5)P_2_ microdomains in coordinating ADBE and SV reformation from bulk endosomes. Details are described in the Discussion section.

ADBE occurs immediately after exocytosis to retrieve required SV protein and lipid constituents to further regenerate SVs under conditions of high-frequency stimulations. Here, we show that the Fwe Ca^2+^ channel-dependent compartmentalization of PI(4,5)P_2_ orchestrates coupling of exocytosis to ADBE and subsequent SV reformation. Based on our findings, we propose a model for this interplay (depicted in Figure 7). Under conditions of strong stimulation, SV exocytosis transfers Fwe from SVs to the periactive zone, where some of the activated Fwe provides the low Ca^2+^ levels that initiate Calcineurin activation to upregulate PI(4,5)P_2_ (Step 1). Increased PI(4,5)P_2_ enhances Fwe Ca^2+^ channel activity, thereby establishing a positive feedback loop that induces PI(4,5)P_2_ microdomain formation (Step 2). High levels of PI(4,5)P_2_ within these microdomains elicit bulk membrane invagination by triggering actin polymerization (Step 3). In parallel, PI(4,5)P_2_ facilitates proper retrieval of Fwe to the bulk endosome (Step 4), thereby terminating the ADBE process. Finally, PI(4,5)P_2_ microdomains dictate SV reformation from the bulk endosomes (Step 5), coordinating ADBE and subsequent SV reformation.

### Fwe-dependent PI(4,5)P_2_ microdomains trigger ADBE

The role of actin polymerization in ADBE has been investigated in mammals (Kononenko et al., 2014; Soykan et al., 2017; Wu et al., 2016), as well as in *Drosophila* (Akbergenova and Bykhovskaia, 2009). PI(4,5)P_2_ is known to control a range of actin regulators, thereby modulating the dynamics of actin polymerization and branching (Janmey et al., 2018). It has been shown previously that, in response to nicotine stimulation, PI(4,5)P_2_ forms clustered microdomains of sub-micrometer scale prior to the appearance of an actin-based ring structure in bovine chromaffin cells (Gormal et al., 2015). In agreement with this observation, we show that intense activity stimulation drives the formation of PI(4,5)P_2_ microdomains at the periactive zone of *Drosophila* NMJ synaptic boutons. Perturbations of the formation of these microdomains reduces ADBE activity very significantly, demonstrating that rapid accumulation of PI(4,5)P_2_ in microdomains is needed to trigger extensive actin polymerization, which likely generates sufficient mechanical force to produce the large endosomes. Furthermore, loss of *fwe* or RNAi-mediated calcineurin knockdown effectively inhibited PI(4,5)P_2_ microdomain formation and, as a consequence, ADBE. Those results are consistent with previous data supporting that Ca^2+^ promotes ADBE by activating its sensor Calcineurin (Cousin and Robinson, 2001; Jin et al., 2019; Sun et al., 2010; Wu et al., 2009; Wu et al., 2014b; Xue et al., 2011).

Our data provide evidence that Fwe-derived Ca^2+^ regulates PI(4,5)P_2_ dynamics through activation of Calcineurin. Phosphatidylinositol 4-phosphate 5-kinases (PIP5Ks) are the major kinases that promote PI(4,5)P_2_ production from precursor phosphoinositides (van den Bout and Divecha, 2009). At mammalian central synapses, PIP5Kγ661, one of the PIP5Kγ isoforms, is the most abundant relative to other isoforms (Wenk et al., 2001). Activation of PIP5Ks can be engaged by binding to its regulators (van den Bout and Divecha, 2009). In response to Ca^2+^ activation, Calcineurin has been shown to dephosphorylate PIP5Kγ661 to promote its interaction with AP-2 complexes, which augments the enzymatic activity of PIP5Kγ661 (Nakano-Kobayashi et al., 2007). Synj is the major PI(4,5)P_2_ phosphatase in neurons (Tsujishita et al., 2001) and it is involved in the SV endocytosis prompted by strong stimulation (Mani et al., 2007). Whereas phosphorylation of Synj by CDK5 inhibits its activity, Calcineurin enhances Synj activity via dephosphorylation (Lee et al., 2004). In yeasts, cells undergo bulk membrane invagination, a process reminiscent of neuronal ADBE, under conditions of hyperosmotic stress (Guiney et al., 2015). However, in that process, Calcineurin dephosphorylates Synj to alter its association with other endocytic partners rather than affecting its enzymatic activity to regulate PI(4,5)P_2_ distribution. Intriguingly, divalent Ca^2+^ ions are known to control the lateral organization of PI(4,5)P_2_, further compartmentalizing PI(4,5)P_2_ into ~70 nm-sized microdomains in monolayers of lipid bilayers via electrostatic interactions between Ca^2+^ and PI(4,5)P_2_ (Carvalho et al., 2008; Ellenbroek et al., 2011; Levental et al., 2009; Sarmento et al., 2014; Wang et al., 2012; Wen et al., 2018). Similarly-sized PI(4,5)P_2_ clusters have been observed in PC12 cells under non-stimulated conditions (van den Bogaart et al., 2011). Therefore, tight spatial and temporal control of the localizations and activities of Fwe, Calcineurin, PIP5K, and Synj may drive Ca^2+^-mediated PI(4,5)P_2_ clustering, perhaps accounting for the Fwe-dependent formation of PI(4,5)P_2_ microdomains at the periactive zone prior to ADBE. Further investigations are needed to characterize the underlying mechanisms.

Our data show that direct binding of PI(4,5)P_2_ is required for the Ca^2+^ channel activity of Fwe. Perturbation of PI(4,5)P_2_-Fwe binding further impaired the formation of PI(4,5)P_2_ microdomains as well as ADBE initiation. Hence, PI(4,5)P_2_ controls Fwe gating, so that Fwe can promote PI(4,5)P_2_ compartmentalization through positive feedback regulation. Furthermore, loss of Fwe impaired the intracellular Ca^2+^ increase that was evoked upon strong activity stimulation (Figure 3g; Yao et al., 2017). These results support that, in addition to PI(4,5)P_2_, the channel function of Fwe may be gated by a significant change in membrane potential. Expanding on that notion, it is therefore possible that both factors may gate Fwe, thereby only allowing channel opening when exocytosis directs Fwe to periactive zones. Future studies should explore the details of this channel gating mechanism.

### PI(4,5)P_2_ microdomains coordinate retrieval of SV membranes and proteins for SV reformation via ADBE

Since ADBE is triggered very rapidly by intense stimuli, it was thought that this type of recycling randomly retrieves SV proteins and that the sorting process takes place when SVs regenerate from bulk endosomes. However, recent work has highlighted a distinct retrieval route for SV proteins during ADBE (Kokotos et al., 2018; Nicholson-Fish et al., 2015). Very little is known about the mechanisms underlying that retrieval route. Interestingly, removing VAMP4 or mutating its di-leucine motif was shown to impair ADBE (Nicholson-Fish et al., 2015). The di-leucine motif of transmembrane proteins is known to mediate binding with the AP-2 adaptor complex (Traub and Bonifacino, 2013). Given that the AP-2 adaptor complex works closely with PI(4,5)P_2_ (McMahon and Boucrot, 2011), these findings imply a role for PI(4,5)P_2_ and the AP-2 adaptor complex in SV protein sorting via ADBE. Indeed, our data show that bulk endosomes recycle few in a PI(4,5)P_2_ microdomain-dependent manner. Hence, in addition to initiating ADBE, PI(4,5)P_2_ may participate in SV protein sorting to bulk endosomes.

SV regeneration occurs following formation of the bulk endosome. Our results also show that either removing Fwe-derived Ca^2+^ or perturbing PI(4,5)P_2_ activity impaired the ability of SVs to reform from the bulk endosome, highlighting the essential role of PI(4,5)P_2_ microdomains in this process. How could PI(4,5)P_2_ of the plasma membrane regulate subsequent SV reformation? It has been shown that PI(4,5)P_2_ is rapidly downregulated on bulk endosomes once formed by ADBE (Chang-Ileto et al., 2011; Cremona et al., 1999; Milosevic et al., 2011). It is conceivable that the high concentrations of PI(4,5)P_2_ in microdomains may compensate for rapid turnover, thereby ensuring appropriate concentrations of PI(4,5)P_2_ or PI(4)P for further recruitment of clathrin and adaptor protein complexes, such as AP-1 and AP-2 (Blumstein et al., 2001; Cheung and Cousin, 2012; Faúndez et al., 1998; Glyvuk et al., 2010; Kokotos et al., 2018; Kononenko et al., 2014; Park et al., 2016). Alternatively, PI(4,5)P_2_ may facilitate SV protein sorting prior to ADBE, meaning proper SV protein compositions on bulk endosomes could control recruitment of adaptor protein complexes. Both of these potential mechanisms are not mutually exclusive and may operate in parallel. Therefore, we propose that the Fwe-dependent formation of PI(4,5)P_2_ microdomains is potentially important in coordinating retrieval of SV membranes and cargos when SVs are recycled via ADBE. Notably, the Fwe channel is evolutionarily conserved from yeast to human (Yao et al., 2009). We have also previously demonstrated conserved functions of Fwe in CME and ADBE at the mammalian central synapse (Yao et al., 2017). A recent study has also identified Fwe as a key protein mediating Ca^2+^-dependent granule endocytosis in mouse cytotoxic T lymphocytes (Chang et al., 2018). Hence, we hypothesize that the mechanism of ADBE we report here may be generally deployed across synapses and species, even in other non-neuronal cells.

## Materials and methods

### *Drosophila* strains and genetics

*fwe* mutants and transgenes: *fwe^DB25^* and *fwe^DB56^* (Yao et al., 2009); *UAS-Flag-Fwe-HA* (Yao et al., 2009); *UAS-Flag-Fwe[E79Q]-HA* (Yao et al., 2017); *UAS-canA1-RNAi(FB4)* (Dijkers and O'Farrell, 2007); *UAS-canA1-RNAi(TRiP.JF01871)* (Bloomington *Drosophila* Stock Center, BDSC#25850); *UAS-synj* (Khuong et al., 2013); *UAS-PLC_δ1_-PH-EGFP* (Verstreken et al., 2009) (Bloomington *Drosophila* Stock Center, BDSC#39693); *UAS-PLC_δ1_-PHS39R-EGFP* (Verstreken et al., 2009) (Bloomington *Drosophila* Stock Center, BDSC#39694); *nSyb-GAL4* (Pauli et al., 2008); *UAS-mCD8-EGFP* (Kyoto Stock Center, DGRC#108068); *vglut-lexA* (Bloomington *Drosophila* Stock Center, BDSC #60314); *13XLexAop2-IVS-GCaMP6f* (Bloomington *Drosophila* Stock Center, BDSC #44277)); *synj^1^* (Verstreken et al., 2003) (Bloomington *Drosophila* Stock Center, BDSC#24883; *LexAop2-PLC_δ1_-PH-APEX2-HA* (This paper); and *LexAop2-PLC_δ1_-PH-EGFP* (This paper). Fly stocks were reared on regular food at 25°C or as otherwise indicated.

### Molecular cloning and transgenesis

For pUAST-HA-Fwe[WT]-APEX2, the coding sequence of the Fwe B isoform and APEX2 were separately PCR-amplified from pUAST-Flag-Fwe-HA (Yao et al., 2009) or pcDNA3 APEX2-NES (addgene #49386), respectively, and then subcloned into the pUAST vector. pUAST-HA-Fwe[K29A/R33A]-APEX2-HA was generated from pUAST-HA-Fwe[WT]-APEX2 through site-directed mutagenesis. pUAST-Flag-Fwe [K29/R33/K95/K100/R105/K146A/K147A/R150A]-HA, pUAST-Flag-Fwe[K146A/K147A/R150A]-HA and pUAST-Flag-Fwe[K95/K100/R105A]-HA were generated from pUAST-Flag-Fwe-HA through site-directed mutagenesis. For plexAop2-PLC_δ1_-PH-APEX2-HA, the coding sequence of the PLC_δ1_-PH domain and APEX2-HA were separately PCR-amplified and then subcloned into pJFRC19-13XLexAop2-IVS-myr:GFP (addgene #26224). For plexAop2-PLC_δ1_-PH-EGFP, the coding sequence of PLC_δ1_-PH-EGFP was PCR-amplified from genomic DNA of the *UAS-PLC_δ1_-PHS39R-EGFP* fly stock and then subcloned into pJFRC19-13XLexAop2-IVS-myr:GFP. For YeMP-Nluc-Fwe-1D4, the cDNA fragment of Nluc and Fwe-1D4 was PCR-amplified from pNL1.1[Nluc] (a gift from Yi-Shiuan Huang) and YeMP-Fwe-1D4 plasmid (Yao et al., 2009), respectively, and then subcloned into the yeast expression YeMP vector. For YeMP-Nluc-Fwe[K29A/R33A]−1D4, YeMP-Nluc-Fwe[K95/K100/R105/K146/K147/R150A]−1D4, and YeMP-Nluc-Fwe[K29/R33/K95/K100/R105/K146/K147/R150A]−1D4, the DNA fragments of the Fwe variant were PCR-amplified from pUAST plasmids and sublconed into the YeMP-Nluc-Fwe-1D4 vector. PCR primers are indicated in key source table (Appendix 1). Transgenic flies were made by Wellgenetics Inc.

### Immunohistochemistry

Third instar larvae were fixed with 4% paraformaldehyde for 20 min. We used 1xPBS buffer containing 0.1% Tween-20 to stain the HA-tagged Fwe proteins. We used 1xPBS buffer containing 0.1% Triton X 100 to stain PLC_δ1_-PH-EGFP or AP-2α. We used 1xPBS buffer containing 0.2% Triton X 100 to stain GCaMP6f. Primary antibodies were used as follows: guinea pig α-Fwe B isoform (1:400) (Yao et al., 2017), chicken α-GFP (Invitrogen, 1:500); mouse α-HA (Sigma, 1:400), mouse α-Bruchpilot (Developmental Studies Hybridoma Bank nc82, 1:100); rabbit α-AP-2α (1:3000) (González-Gaitán and Jäckle, 1997) guinea pig α-Eps15 (1:3000) (Koh et al., 2007); rabbit α-HRP conjugated with Alexa Fluor 488, Cy3 or Cy5 (Jackson ImmunoResearch Laboratories, 1:250). Secondary antibodies conjugated to Alexa Fluor 488, Alexa Fluor 555, or Alexa Fluor 647 (Invitrogen and Jackson ImmunoResearch) were used at 1:500. The NMJ boutons were derived from muscles 6 and 7 of abdominal segments 2/3. To detect PI(4,5)P_2_ microdomains, the NMJ boutons were fixed immediately after high K^+^ stimulation. To quantitatively compare PLC_δ1_-PH-EGFP or AP-2α immunostaining signal among different sets of experiments, fixed larval fillets derived from different conditions were collected into the same Eppendorf tube. The NMJ boutons were stained for PLC_δ1_-PH-EGFP using α-GFP and for the neuronal membranes using fluorescein-conjugated α-HRP. Consecutive single-plane images of the boutons of muscles 6 and 7 in abdominal segments 2 or 3 of all different experimental sets were taken using a Zeiss LSM 780 confocal microscope with a Plan-Apochromat 63x/1.4 Oil DIC M27 objective under a 1 μm interval setup and equal laser power and laser exposure time. For data quantification, single-plane images of five different individual boutons from each NMJ bouton image were used. The presynaptic plasma membrane regions of the type Ib boutons defined by α-HRP immunostaining were outlined manually, and native fluorescence or α-GFP immunostaining signal intensities of PLC_δ1_-PH-EGFP or AP-2α on the plasma membrane were quantified using ImageJ and averaged to serve as one individual data-point. For data quantification of SIM images, we chose single-plane SIM images focused on a central section of an individual bouton and outlined plasma membrane-associated PLC_δ1_-PH-EGFP-immunostained clusters using ImageJ. The area of these clusters of each individual bouton was measured using ImageJ, and the areas of clusters measuring over 0.032 μm^2^ (equal to a ~ 200 nm diameter circle, i.e. the resolution limit of SIM) were averaged to serve as one data-point. We assessed 37 boutons derived from five NMJs of three different larvae. Image processing was achieved using LSM Zen.

### Western blot

For western blotting, the brain and ventral nerve chord of larval fillets were removed and subjected to different stimulation conditions. Afterwards, the fillets were crushed in 1xSDS sample buffer and boiled for 5 min. Dilutions for primary antibodies were as follows: mouse anti-α-actin, 1:20000 (Sigma); chicken anti-GFP, 1:5000 (Invitrogen).

### PLA

Third-instar larvae were fixed with 4% paraformaldehyde for 20 min and permeabilized with 1xPBS buffer containing 0.1% Tween-20. Larval fillets were incubated with mouse α-HA (Sigma, 1:200) and rabbit α-GFP (Invitrogen, 1:500) in 1xPBS buffer containing 0.1% Tween-20 at 4°C for 12 hr. Excess antibodies were washed out using 1xPBS buffer containing 0.1% Tween-20. The samples were mixed with the PLA probe (Sigma, 1:5) for 2 hr at 37°C. After washing with 1x buffer A, the samples were incubated with ligation solution (1:40) for 1.5 hr at 37°C. After again washing with 1x buffer A, the samples were incubated with amplification solution (1:80) for 2 hr at 37°C. Next, the samples were washed with 1x buffer B and then 0.01x buffer B. The samples were stained with anti-chicken Alexa Fluor 488-conjugated IgG and anti-mouse Alexa Fluor 647-conjugated IgG, followed by a wash of 1x PBS buffer containing 0.1% Tween-20. To quantitatively compare PLA signal, fixed larval fillets derived from different experimental conditions were collected into the same Eppendorf tube and processed. Consecutive single-plane images of the boutons of muscles 6 and 7 in abdominal segments 2 or 3 of all different experimental sets were taken using a Zeiss LSM 780 confocal microscope with a Plan-Apochromat 63x/1.4 Oil DIC M27 objective under a 1 μm interval setup and equal laser power and laser exposure time. For data quantification, consecutive Z-plane images spanning whole NMJ were projected under maximal fluorescence intensity. All type Ib boutons in individual Z-projection image were outlined according to PLC_δ1_-PH-EGFP-stained regions. PLA or antibody immunostaining signal intensities within the boutons and background staining signals in surrounding muscles were counted using ImageJ and averaged. One individual data-point was obtained by muscular background signal subtraction. Image processing was achieved using LSM Zen.

### Nluc-Fwe-1D4 purification and BRET assay

The Nluc-Fwe-1D4 fusion protein was purified as described previously (Yao et al., 2009). Briefly, plasma membrane was isolated from a two liter culture of yeast strain BJ5457 expressing Nluc-Fwe-1D4 protein. The plasma membranes were solubilized at 4°C with 10x critical micelle concentration (CMC) DDM (Anatrace) in a solution of 20 mM HEPES (pH8.0), 300 mM NaCl, 10% Glycerol, 2.0 mM DTT, and 1 mM PMSF for 2 hr. Insoluble membranes were spun down by centrifugation at 100,000 ×*g* for 60 min. The lysates including solubilized Nluc-Fwe-1D4 proteins were cleaned with CNBr sepharose 4B at 4°C for 1 hr. The samples were then mixed with α−1D4-conjugated CNBr-activated Sepharose 4B at 4°C for 8–12 hr. After washing with a solution of 20 mM HEPES (pH8.0), 150 mM NaCl, 10% Glycerol, 2.0 mM DTT, 1 mM PMSF and 2.6xCMC DDM, the protein was eluted with a 1D4 peptide-containing buffer (3 mg of 1D4 peptide in 1 ml of washing buffer). Purified proteins were subjected to SDS-PAGE and detected using Lumitein staining or western blotting with anti-α−1D4 antibody at 1:5000 (Yao et al., 2009). The BRET assay was performed in a 384-well plate, with each well containing 30 μl of reaction solution [0.5 nM purified proteins, 5 μM BODIPY-TMR Phosphatidylinositol 4,5-bisphosphate (C-45M16A, Echelon Bioscience), furimazine (Promega, 1:2000), 20 mM HEPES, 150 mM NaCl, 10% Glycerol, 2 mM DTT, 1 mM PMSF, 4 mM DDM]. Fluorescence signal was detected using a Microplate Reader M1000 pro (Tecan) with two different emission spectrum filters, that is 500–540 nm for Nluc and 550–630 nm for BODIPY-TMR Phosphatidylinositol 4,5-bisphosphate. For competition assay, 30 μl of the reaction solution was included with 1 mM brain phosphatidylinositol 4,5-bisphosphate (Avanti). The BRET signal was calculated according to the following formula:

### Live imaging

For PLC_δ1_-PH-EGFP imaging, third-instar larvae were dissected in a zero-calcium HL-3 solution at room temperature. For groups stimulated with electric pulses, larval fillets were bathed in a solution of 2 mM Ca^2+^ (70 mM NaCl, 5 mM KCl, 10 mM MgCl_2_, 10 mM NaHCO_3_, 5 mM trehalose, 5 mM HEPES (pH 7.4), 115 mM sucrose, 2 mM CaCl_2_). High concentrations of glutamate were used to desensitize glutamate receptors, thereby reducing muscle contraction when stimulated. A cut axonal bundle was sucked into the tip of a glass capillary electrode and then stimulated at 20 or 40 Hz for 3 min. Stimulus strength was set at 5 V and 0.5 ms duration by means of pClamp 10.6 software (Axon Instruments Inc). Ten images were taken from larval fillets at rest. After stimulation for 2 min, muscle contraction significantly decelerated. Thus, we captured 60 consecutive snapshot images every second from the third minute. Muscles 6 and 7 of abdominal segment three were imaged. Under the condition of high K^+^ stimulation, larval fillets were bathed in a solution of 90 mM K^+^/2 mM Ca^2+^/7 mM glutamate (25 mM NaCl, 90 mM KCl, 10 mM MgCl_2_, 10 mM NaHCO_3_, 5 mM trehalose, 5 mM HEPES (pH 7.4), 30 mM sucrose, 2 mM CaCl_2_, 7 mM monosodium glutamate) for 5 min. Sixty consecutive snapshot images were captured every second from the fifth minute of stimulation. Images were taken using a long working distance water immersion objective (XLUMPLFLN20XW, Olympus) and EMCCD camera (iXon, Andor) mounted on a SliceScope Pro 6000 (Scientifica) microscope and employing MetaFluor software (Molecular Devices). For GCaMP6f imaging, third instar larvae were dissected in a zero-calcium HL-3 solution at room temperature. Ten images were taken from larval fillets at rest. Subsequently, larval fillets were stimulated with a solution of 90 mM K^+^/2 mM Ca^2+^/7 mM glutamate for 5 min. Sixty consecutive snapshot images were captured every second from the fifth minute of stimulation. The *lexA/lexAop2* binary system was used to stably express a comparable level of GCaMP6f in the presynaptic compartment of NMJ boutons for all tested genotypes, allowing us to compare Ca^2+^ imaging results when the resting Ca^2+^ levels were potentially affected by differences in genetic background. We stained for the GCaMP6f protein using a-GFP antibody and confirmed comparable GCaMP6f levels among the different genotypes tested in each dataset. Evoked Ca^2+^ levels were calculated by subtracting the resting GCaMP6f fluorescence from the GCaMP6f fluorescence induced by high K^+^ stimulation. The NMJs were derived from muscles 6 and 7 of abdominal segment 2/3. Images were taken using a water immersion objective (W Plan-Apochromat 40x/1.0 DIC M27, Zeiss). For each imaging experiment, at least three focused images for the same boutons under resting, stimulation, or post-stimulation conditions were used for data quantification. Fluorescence intensities of PLC_δ1_-PH-EGFP or GCaMP6f within the boutons were quantified using ImageJ and averaged to serve as one individual data-point. Image processing was achieved using LSM Zen.

### Transmission electron microscopy

Third instar larval fillets were prepared in zero-calcium HL-3 solution at room temperature. For the resting conditions, the fillets were bathed in zero-calcium HL-3 solution at room temperature for another 10 min before fixation. For the high K^+^ stimulation conditions, fillets were bathed in a solution of 90 mM K^+^ and 2 mM Ca^2+^ for 10 min. The stimulation was terminated by washing three times with zero-calcium HL-3 solution, followed by fixation. For recovery conditions, following high K^+^ stimulation, fillets were bathed in zero-calcium HL-3 solution at room temperature for 10 or 20 min before fixation. Larval fillets were fixed for 12 hr at 4°C in 4% paraformaldehyde/1% glutaraldehyde/0.1 M cacodylic acid (pH 7.2), rinsed with 0.1 M cacodylic acid (pH 7.2), and postfixed with 1% OsO_4_ and 0.1 M cacodylic acid at room temperature for 3 hr. These samples were then subjected to a series of dehydration steps using 30–100% ethanol. After 100% ethanol dehydration, the samples were sequentially incubated with propylene, a mixture of propylene and resin, and pure resin. Finally, the samples were embedded in 100% resin. TEM images were captured using Tecnai G2 Spirit TWIN (FEI Company) and a Gatan CCD Camera (794.10.BP2MultiScanTM). NMJ boutons were captured at high magnifications. For each condition, NMJ bouton images were taken from at least five different NMJs of each third-instar larvae, and three to five larvae were used. Quantifications were performed using ImageJ. For diaminobenzidine (DAB) polymerization, third instar larvae were dissected at room temperature in zero-calcium HL-3 medium, followed by a 10 min incubation in 5 mM K^+^/0 Ca^2+^ mM solution or a 10 min stimulation of 90 mM K^+^/2 mM Ca^2+^. Next, the samples were subjected to 30 min fixation in ice-cold 4% paraformaldehyde/1% glutaraldehyde/0.1 M cacodylic acid (pH 7.2). Subsequently, the samples were transferred to Eppendorf tubes for 15 min incubation with a solution of 0.5 mg/ml DAB solution, followed by incubation with a solution of 0.5 mg/ml DAB and 0.006% H_2_O_2_ for 15 min at room temperature. This latter step was repeated once to ensure DAB polymerization. Samples were washed three times with 1xPBS buffer for 10 min and then fixed with a solution of 4% paraformaldehyde/1% glutaraldehyde/0.1 M cacodylic acid (pH 7.2) for 12 hr at 4°C, followed by fixation with a solution of 1% OsO_4_/0.1 M cacodylic acid at room temperature for 3 hr. Then, standard dehydration, embedding, and imaging were performed. For data quantifications of DAB intensities, the display color of TEM images was reverted to grayscale using ImageJ. Average DAB staining intensity on each individual bulk endosome was quantified. Then, the average DAB staining intensity on 50–100 surrounding SVs from the same bouton image was used to assess the relative level of HA-Fwe-APEX2 on bulk endosomes vs SVs.

### Statistics

All data analyses were conducted using GraphPad Prism 8.0, unless stated otherwise. Paired and multiple datasets were compared by Student *t*-test or one-way ANOVA with Tukey’s post hoc test, respectively. Individual data values are biological replicates. Samples were randomized during preparation, imaging, and data processing to minimize bias.

## Data Availability

All data generated or analysed during this study are included in the manuscript and supporting files. Source data files have been provided.

## Additional files

Transparent reporting form

## Appendix 1

### key resources table

**Appendix 1—key resources table:**

Reagent type (species) or resource	Designation	Source or reference	Identifiers	Additional information
Genetic reagent (*D. melanogaster*)	*fwe^DB25^*	Yao et al., 2009		*fwe* mutant allele used in Figures 3–6, Figure 4—figure supplements 1–2, Figure 5—figure supplement 1, and Figure 6—figure supplement 1
Genetic reagent (*D. melanogaster*)	*fwe^DB56^*	Yao et al., 2009		*fwe* mutant allele used in Figures 3–6, Figure 4—figure supplements 1–2, Figure 5—figure supplement 1, and Figure 6—figure supplement 1
Genetic reagent (*D. melanogaster*)	*UAS-Flag-Fwe-HA*	Yao et al., 2017		Wild-type version of *fwe* transgene used in Figure 3a–b, Figure 6a, Figure 4—figure supplement 1, and Figure 6—figure supplement 1b
Genetic reagent (*D. melanogaster*)	*UAS-Flag-Fwe[E79Q]-HA*	Yao et al., 2017		Channel deficient version of *fwe* transgene used in Figure 3e–f
Genetic reagent (*D. melanogaster*)	*UAS-canA1-RNAi (FB4)*	Dijkers and O'Farrell, 2007		RNAi line of *canA1* used in Figure 3g–j
Genetic reagent (*D. melanogaster)*	*UAS-canA1-RNAi(TRiP.JF01871)*	Bloomington *Drosophila* Stock Center Dijkers and O'Farrell, 2007	RRID:BDSC_25850	RNAi line of *canA1* used in Figure 3g–h, 5
Genetic reagent (*D. melanogaster*)	*UAS-synj*	Khuong et al., 2013		Wild-type version of *synj* transgene used in Figure 2b–d, Figure 3i–j, Figure 6d–f, and Figure 2—figure supplement 1c.
Genetic reagent (*D. melanogaster*)	*UAS-PLC_δ1_-PH-EGFP*	Bloomington *Drosophila* Stock Center Verstreken et al., 2009	RRID:BDSC_39693	PI(4,5)P_2_ reporter transgene used in Figures 1–4, Figure 1—figure supplement 1, Figure 1—figure supplement 3c, Figure 2—figure supplements 1–2, Figure 4—figure supplement 1e–f.
Genetic reagent (*D. melanogaster*)	*UAS-PLC_δ1_-PHS39R-EGFP*	Bloomington *Drosophila* Stock Center Verstreken et al., 2009	RRID:BDSC_39694	PI(4,5)P_2_-binding mutant transgene used in Figure 2c–d, Figure 1—figure supplement 2c–e
Genetic reagent (*D. melanogaster*)	*nSyb-GAL4*	Bloomington *Drosophila* Stock Center Pauli et al., 2008	RRID:BDSC_51635	Neuronal *GAL4* driver used in all figures
Genetic reagent (*D. melanogaster*)	*vglut-lexA*	Bloomington *Drosophila* Stock Center	RRID:BDSC_60314	Motor neuron *lexA* driver used in Figure 3e–f, Figure 3i–j, Figure 4d–e, and Figure 4—figure supplement 1c–d
Genetic reagent (*D. melanogaster*)	*13XLexAop2-IVS-GCaMP6f*	Bloomington *Drosophila* Stock Center	RRID:BDSC_44277	Ca^2+^ indicator transgene used in Figure 3i–j, Figure 4d–e, and Figure 4—figure supplement 1c–d
Genetic reagent (*D. melanogaster*)	*synj^1^*	Bloomington *Drosophila* Stock Center Verstreken et al., 2003	RRID:BDSC_24883	*synj* mutant allele used in Figure 1—figure supplement 1c–d
Genetic reagent (*D. melanogaster*)	*UAS-mCD8-EGFP*	Kyoto Stock Center	RRID:DGRC_108068	*mCD8-EGFP* transgene used in Figure 1—figure supplement 2a–b
Genetic reagent (*D. melanogaster*)	*UAS-HA-Fwe[WT]-APEX2*	This paper		APEX2 fusion version of wild-type *fwe* transgene used in Figure 4d–g, Figure 5, Figure 6b–f, Figure 4—figure supplement 2, and Figure 6—figure supplement 1c–d
Genetic reagent (*D. melanogaster*)	*UAS-HA-Fwe[K29A/R33A]-APEX2*	This paper		APEX2 fusion version of *fwe[K29A/R33A]* transgene used in Figure 4d–g, Figure 5, Figure 4—figure supplement 2
Genetic reagent (*D. melanogaster*)	*UAS-Flag-Fwe[K29A/R33A/K95A/K100A/R105A/K146A/K147A/R150A]-HA*	This paper		*fwe[K29A/R33A/K95A/K100A/R105A/K146A/K147A/R150A]* transgene was not expressed stably
Genetic reagent (*D. melanogaster*)	*UAS-Flag-Fwe[K146A/K147A/R150A]-HA*	This paper		*fwe[K146A/K147A/R150A]* transgene used in Figure 4—figure supplement 1
Genetic reagent (*D. melanogaster*)	*UAS-Flag-Fwe[K95A/K100A/R105A]-HA*	This paper		*fwe[K95A/K100A/R105A]* transgene was not expressed stably
Genetic reagent (*D. melanogaster*)	*LexAop2-PLC_δ1_-PH-APEX2-HA*	This paper		APEX2 fusion version of PLCδ1-PH transgene used in Figure 3i–j
Genetic reagent (*D. melanogaster*)	*LexAop2-PLCδ1-PH-EGFP*	This paper		*lexAop2* version of PLC_δ1_-PH-EGFP transgene used in Figure 3e–f
Antibody	α-GFP (Chicken polyclonal)	Invitrogen	Cat #A10262, RRID:AB_2534023	IF: 1:500 WB: 1:5000
Antibody	α-HA (Mouse monoclonal)	Sigma	Cat # H3663, RRID:AB_262051	IF: 1:400 PLA: 1:200
Antibody	α-Bruchpilot (Mouse monoclonal)	DSHB Wagh et al., 2006	Cat# nc82, RRID:AB_2314866	IF: 1:100
Antibody	α-AP-2α (Rabbit polyclonal)	González-Gaitán and Jäckle, 1997		IF: 1:3000
Antibody	α-Eps15 (Guinea pig polyclonal)	Koh et al., 2007		IF: 1:3000
Antibody	α-Fwe B isoform (Guinea pig polyclonal)	Yao et al., 2017		IF: 1:400
Antibody	Cy3 AffiniPure Rabbit Anti-Horseradish Peroxidase	Jackson ImmunoResearch Labs	Cat# 323-165-021, RRID:AB_2340262	IF: 1:250
Antibody	Alexa Fluor 488 AffiniPure Rabbit Anti-Horseradish Peroxidase	Jackson ImmunoResearch Labs	Cat# 323-545-021, RRID:AB_2340264	IF: 1:250
Antibody	α-GFP (Rabbit polyclonal)	Thermo Fisher Scientific	Cat# A-6455, RRID:AB_221570	PLA: 1:500
Antibody	α-actin (Mouse monoclonal)	Sigma	Cat# A7732, RRID:AB_2221571	WB:1:20000
Antibody	α−1D4 (Mouse monoclonal)	Yao et al., 2009		WB: 1:2000
Strain, strain background (*Escherichia coli*)	DH5α			
Strain, strain background (*Escherichia coli*)	yeast strain BJ5457	Yao et al., 2009		
Peptide, recombinant protein	Nluc-Fwe-1D4 fusion protein	This paper		Used in Figure 4b–c
Peptide, recombinant protein	Nluc-Fwe[K29A/R33A]−1D4	This paper		Used in Figure 4b–c
Peptide, recombinant protein	Nluc-Fwe[K95A/K100A/R105A/K146A/K147/R150A]−1D4	This paper		Used in Figure 4b–c
Peptide, recombinant protein	Nluc-Fwe[K29A/R33A/K95A/K100A/R105A/K146A/K147A/R150A]−1D4	This paper		Used in Figure 4b–c
Chemical compound, drug	n-Dodecyl-β-D-Maltopyranoside	Anatrace	Cat# D310	Used in Figure 4b–c
Chemical compound, drug	BODIPY-TMR Phosphatidylinositol 4,5-bisphosphate	Echelon Bioscience	Cat#C-45M16A	Used in Figure 4b–c
Chemical compound, drug	Furimazine	Promega	Cat# N1110	Used in Figure 4b–c
Chemical compound, drug	Brain Phosphatidylinositol 4,5-bisphosphate	Avanti Polar Lipids	AV-840046P	Used in Figure 4b–c
Commercial assay or kit	Duolink In Situ Red Starter Kit Mouse/Rabbit	Sigma	Cat# DUO92101	Used in Figure 3a–b
Commercial assay or kit	NEBuilder HiFi DNA Assembly Master Mix	NEB	Cat# E2621	
Recombinant DNA reagent	YeMP	Yao et al., 2009		
Recombinant DNA reagent	pcDNA3 APEX2-NES	Addgene	RRID:Addgene_49386	
Recombinant DNA reagent	pJFRC19-13XLexAop2-IVS-myr:GFP	Addgene	RRID:Addgene_26224	
Recombinant DNA reagent	pNL1.1[Nluc]	Promega	Cat# #N1001	
Recombinant DNA reagent	pUAST-HA-Fwe[WT]-APEX2	This paper		DNA construct of APEX2 fusion version of wild-type fwe transgene used in Figure 4d–g, Figure 5, Figure 6b–f, Figure 4—figure supplement 2, and Figure 6—figure supplement 1c–d
Recombinant DNA reagent	pUAST-HA-Fwe[K29A/R33A]-APEX2	This paper		DNA construct of APEX2 fusion version of *fwe[K29A/R33A]* transgene used in Figure 4d–g, Figure 5, Figure 4—figure supplement 2
Recombinant DNA reagent	pUAST-Flag-Fwe-HA	Yao et al., 2017		
Recombinant DNA reagent	pUAST-Flag-Fwe[K29/R33/K95/K100/R105/K146A/K147A/R150A]-HA	This paper		DNA construct of *fwe[K29A/R33A/K95A/K100A/R105A/K146A/K147A/R150A]* transgene
Recombinant DNA reagent	pUAST-Flag-Fwe[K146A/K147A/R150A]-HA	This paper		DNA construct of *fwe[K146A/K147A/R150A]* transgene used in Figure 4—figure supplement 1
Recombinant DNA reagent	pUAST-Flag-Fwe[K95/K100/R105A]-HA	This paper		DNA construct of *fwe[K95A/K100A/R105A]* transgene
Recombinant DNA reagent	plexAop2-PLC_δ1_-PH-APEX2-HA	This paper		DNA construct of APEX2 fusion version of PLCδ1-PH transgene used in Figure 3i–j
Recombinant DNA reagent	plexAop2-PLC_δ1_-PH-EGFP	This paper		DNA construct of *lexAop2* version of PLC_δ1_-PH-EGFP transgene used in Figure 3e–f
Recombinant DNA reagent	YeMP-Nluc-Fwe-1D4	This paper		DNA construct of expression of Nluc-Fwe-1D4 recombinant protein used in Figure 4b–c
Recombinant DNA reagent	YeMP-Nluc-Fwe[K29A/R33A]−1D4	This paper		DNA construct of expression of Nluc-Fwe[K29A/R33A]−1D4 recombinant protein used in Figure 4b–c
Recombinant DNA reagent	YeMP-Nluc-Fwe[K95A/K100A/R105A/K146A/K147/R150A]−1D4	This paper		DNA construct of expression of Nluc-Fwe[K95A/K100A/R105A/K146A/K147/R150A]−1D4 recombinant protein used in Figure 4b–c
Recombinant DNA reagent	YeMP-Nluc-Fwe[K29A/R33A/K95A/K100A/R105A/K146A/K147A/R150A]−1D4	This paper		DNA construct of expression of Nluc-Fwe[K29A/R33A/K95A/K100A/R105A/K146A/K147A/R150A]−1D4-1D4 recombinant protein used in Figure 4b–c
Sequence-based reagent	HA-Fwe[WT]-APEX2 forward-1	This paper		5'- tcgttaacagatcttGCGGCCGCATGTACCCATACGATGTTCCAGATTACG-3'
Sequence-based reagent	HA-Fwe[WT]-APEX2 Reverse-1	This paper		5'- tgaacctcctccgccTGTGGGGCGCCAGACATC-3'
Sequence-based reagent	HA-Fwe[WT]-APEX2 forward-2	This paper		5'- gccccacaggcggaggaggttcaggaggcggaggttcgGGTACCGGCGGAAAGAGTTACCCGAC-3'
Sequence-based reagent	HA-Fwe[WT]-APEX2 Reverse-2	This paper		5'- tccttcacaaagatccTCTAGAGGCGTCGGCGAATCCCAG −3'
Sequence-based reagent	HA-Fwe[K29A/R33A]-APEX2-HA forward	This paper		5'-CAGCCGTGGTATCTCGCATATGGAAGTGCATTGCTGGGCATTG-3'
Sequence-based reagent	Flag-Fwe[K95/K100/R105A]-HA forward	This paper		5'-GGAATCTGCGCCCTTGTACTTCGCTGCCGGGCTCTACATTGCCATGGCCATTCCGCCCATTATT-3'
Sequence-based reagent	Flag-Fwe[K146A/K147A/R150A]-HA forward	This paper		5'-CAGAGGACATGGCCGCCGCTGCCACATCGCCCACACAGATGGCCGGCAGTCAGGCGGGCGG-3'
Sequence-based reagent	PLC_δ1_-PH-APEX2-HA forward-1	This paper		5'-tcttatcctttacttcagGCGGCCGCATGGACTCGGGCCGGGAC-3'
Sequence-based reagent	PLC_δ1_-PH-APEX2-HA forward-2	This paper		5'- GCTGTACAAGggcggaggaggttcaggaggcggaggttcgGGCGGAAAGAGTTACCCGAC-3'
Sequence-based reagent	PLC_δ1_-PH-APEX2-HA reverse-1	This paper		5'- cctccgccggtaccAAGATCTTCCGGGCATAGCTGTCG-3'
Sequence-based reagent	PLC_δ1_-PH-APEX2-HA reverse-2	This paper		5'- ggttccttcacaaagatcctctagattaagcgtaatctggaacatcgtatgggtaGGCGTCGGCGAATCCCAG −3'
Sequence-based reagent	PLC_δ1_-PH-EGFP forward	This paper		5'- tcttatcctttacttcagGCGGCCGCATGGACTCGGGCCGGGAC-3'
Sequence-based reagent	PLC_δ1_-PH-EGFP reverse	This paper		5'- TTTCTAGATTACTTGTACAGCTCGTCCAT-3'
Sequence-based reagent	YeMP-Nluc-Fwe-1D4 forward-1	This paper		5'- CCACTAGTATGGTCTTCACACTCGAAG-3'
Sequence-based reagent	YeMP-Nluc-Fwe-1D4 forward-2	This paper		5'- AAAACTAGTGTCGACTCGTTTGCGGAAAAGATAACG −3'
Sequence-based reagent	YeMP-Nluc-Fwe-1D4 Reverse-1	This paper		5'-AAAGTCGACCGAACCTCCGCCTCC-3'
Sequence-based reagent	YeMP-Nluc-Fwe-1D4 Reverse-2	This paper		5'- AAAACGCGTTTACGCAGGCGCGACTTGG-3'
Sequence-based reagent	YeMP-Nluc-Fwe[K29A/R33A]−1D4, YeMP-Nluc-Fwe[K95A/K100A/R105A/K146A/K147/R150A]−1D4, YeMP-Nluc-Fwe[K29A/R33A/K95A/K100A/R105A/K146A/K147A/R150A]−1D4 forward	This paper		5'- AAAACTAGTGTCGACTCGTTTGCGGAAAAGATAACG-3'
Sequence-based reagent	YeMP-Nluc-Fwe[K29A/R33A]−1D4, YeMP-Nluc-Fwe[K95A/K100A/R105A/K146A/K147/R150A]−1D4, YeMP-Nluc-Fwe[K29A/R33A/K95A/K100A/R105A/K146A/K147A/R150A]−1D4 Reverse	This paper		5'- AAAACGCGTTTACGCAGGCGCGACTTGGCTGGTCTCTGTTGTGGGGCGCC-3'
Software, algorithm	LSM Zen	Zeiss	https://www.zeiss.com/microscopy/us/products/microscope-software/zen-lite.html	
Software, algorithm	Image J	https://imagej.net/contributors	https://imagej.nih.gov/ij/	
Software, algorithm	pClamp 10.6	Moleculardevices	https://www.moleculardevices.com/products/cellular-imaging-systems/acquisition-and-analysis-software/metamorph-microscopy#gref	
Software, algorithm	MetaMorph	Moleculardevices	https://www.moleculardevices.com/products/cellular-imaging-systems/acquisition-and-analysis-software/metamorph-microscopy#gref	
Software, algorithm	GraphPad Prism 8.0	Prism	https://www.graphpad.com/	
